# OpgH is an essential regulator of *Caulobacter* morphology

**DOI:** 10.1128/mbio.01443-24

**Published:** 2024-08-15

**Authors:** Allison K. Daitch, Erika L. Smith, Erin D. Goley

**Affiliations:** 1 Department of Biological Chemistry, Johns Hopkins University School of Medicine, Baltimore, Maryland, USA; Fred Hutchinson Cancer Center, Seattle, Washington, USA

**Keywords:** *Caulobacter*, morphology, osmoregulated periplasmic glucans, OpgH, divisome, elongasome, peptidoglycan, CenKR, two-component regulatory systems

## Abstract

Bacterial growth and division rely on intricate regulation of morphogenetic complexes to remodel the cell envelope without compromising envelope integrity. Significant progress has been made in recent years towards understanding the regulation of cell wall metabolic enzymes. However, other cell envelope components play a role in morphogenesis as well. A primary factor required to protect envelope integrity in low osmolarity environments is OpgH, the synthase of osmoregulated periplasmic glucans (OPGs). Here, we demonstrate that OpgH is essential in the α-proteobacterium *Caulobacter crescentus*. Unexpectedly, depletion of OpgH or attempted complementation with a catalytically dead OpgH variant results in striking asymmetric bulging and cell lysis. These shape defects are accompanied by reduced cell wall synthesis and mislocalization of morphogenetic complexes. Interestingly, overactivation of the CenKR two-component system that has been implicated in cell envelope stress homeostasis in α-proteobacteria phenocopies the morphogenetic defects associated with OpgH depletion. Each of these perturbations leads to an increase in the levels of the elongasome protein, MreB, and decreases in the levels of divisome proteins FtsZ and MipZ as well as OpgH, itself. Constitutive production of OpgH during CenKR overactivation prevents cell bulging, but cells still exhibit morphogenetic defects. We propose that OPG depletion activates CenKR, leading to changes in the expression of cell envelope-related genes, but that OPGs also exert CenKR-independent effects on morphogenesis. Our data establish a surprising function for an OpgH homolog in morphogenesis and reveal an essential role of OpgH in maintaining cell morphology in *Caulobacter*.

**IMPORTANCE:**

Bacteria must synthesize and fortify the cell envelope in a tightly regulated manner to orchestrate growth and adaptation. Osmoregulated periplasmic glucans (OPGs) are important, but poorly understood, constituents of Gram-negative cell envelopes that contribute to envelope integrity and protect against osmotic stress. Here, we determined that the OPG synthase OpgH plays a surprising, essential role in morphogenesis in *Caulobacter crescentus*. Loss of OpgH causes asymmetric cell bulging and lysis via misregulation of the localization and activity of morphogenetic complexes. Overactivation of the CenKR two-component system involved in envelope homeostasis phenocopies OpgH depletion, suggesting that depletion of OpgH activates CenKR. Because cell envelope integrity is critical for bacterial survival, understanding how OpgH activity contributes to morphogenesis and maintenance of envelope integrity could aid in the development of antibiotic therapies.

## INTRODUCTION

Bacteria inhabit an impressive range of environments and can adapt to sudden changes in environmental conditions. One parameter that can vary significantly in different niches is osmolarity, ranging from dilute freshwater habitats to highly concentrated soil. The α-proteobacterium, *Caulobacter crescentus* (hereafter *Caulobacter*), is well-established as a model for physiological adaptation in the face of changing environments. Originally classified as an aquatic oligotroph, there is now evidence that *Caulobacter* also inhabits soil environments (1). Within these diverse habitats, *Caulobacter* is capable of exquisitely tuning its physiology as needed to propagate through growth and division.

The surrounding environment and available nutrients dictate *Caulobacter* cell cycle progression (2). *Caulobacter* undergoes distinct morphological changes as it proceeds through its cell cycle. A newborn, flagellated swarmer cell has the ability to move and search for nutrients before differentiating into a sessile stalked cell (2). This transition involves a stereotyped set of physiological changes including shedding its flagellum and growing a thin extension of the cell envelope called the stalk (2). The stalked cell can then undergo a cycle of cell division, as characterized by replication and segregation of the chromosome, elongation of the cell body, growth of a flagellum opposite the stalked pole, and cytokinesis (2). This asymmetric life cycle enables swarmer cells to find a new environment while requiring stalked cells to adapt within a given environment and any changes they experience there.

*Caulobacter* has distinct cellular structures and processes that allow for its survival in changing environments. Notably, the bacterial cell envelope serves as the physical barrier between the cell and its environment (3). The Gram-negative cell envelope comprises the inner and outer cell membranes and the periplasm between them. Within the periplasm is the peptidoglycan (PG) cell wall which provides structure and shape to the cell and protects the cell from lysis due to turgor pressure (4). PG biosynthesis is coordinated largely by the localization and activity of two conserved morphogenetic complexes: the elongasome/Rod complex and the divisome, responsible for elongation and division, respectively (5). Without the cell wall, significant reduction in osmolarity would quickly alter cell shape and result in lysis.

In addition to the cell wall, some bacteria also produce glucose polymers in the periplasm, called osmoregulated periplasmic glucans (OPGs, also referred to as membrane-derived oligosaccharides). In *Escherichia coli*, OPGs increase in abundance in low osmolarity environments and are proposed to function as osmoprotectants (6–8). Theoretically, OPGs modulate the osmolarity of the periplasm to protect the cytoplasmic osmolarity from environmental changes. OPGs are produced across proteobacteria, though their structures can vary significantly, ranging from 5 to 24 glucose units in linear or cyclic configurations, and sometimes bearing modifications from phospholipids or intermediary metabolism (6, 7). We recently identified the first OPG in *Caulobacter*: a cyclic hexamer of glucose (9).

The synthase of OPGs has been characterized in other organisms as the inner membrane protein, OpgH. In *E. coli*, OpgH is necessary for OPG production (8). Some organisms, including *E. coli*, encode additional *opg* genes, such as *opgG and opgD*, which are postulated to modify OPGs (6, 10–12). Surprisingly, however, the only *opg* homolog encoded in *Caulobacter* is *opgH* (*CCNA_02097*). Also notably, *Caulobacter opgH* is annotated as essential (13), unlike characterized *opgH* homologs in other organisms. OpgH and other OPG synthetic enzymes have been functionally implicated in osmoprotection, antibiotic resistance, motility, virulence, and symbiosis, but have not been reported to be essential for viability in any organism studied thus far (14).

In this work, we explore the role of OpgH in *Caulobacter* growth and morphogenesis. We demonstrate the essentiality of *opgH* in *Caulobacter* and discover striking morphological defects associated with OpgH depletion or loss of OpgH enzymatic activity. These unique morphological defects phenocopy overactivation of the CenKR two-component system that is involved in cell envelope homeostasis (15). Our data reveal a novel role for an OpgH homolog, and OPGs, in maintaining cell morphology under unstressed growth conditions and provide a putative connection between OpgH activity and CenKR activation.

## RESULTS

### OpgH is essential in *Caulobacter*

This study was initiated through our interest in identifying essential components of the cell envelope that contribute to morphogenesis. Transposon sequencing indicated that *opgH* (*CCNA_02097*), encoding a putative inner membrane-associated glucan glycosyltransferase, was essential in *Caulobacter* (13). This was surprising because in *E. coli*, *opgH* is non-essential and deletion of *opgH* yields minimal defects in unstressed conditions (16). We therefore sought to validate the predicted essentiality of *opgH* in *Caulobacter*. Indeed, we were unable to generate a deletion of *opgH*. We were, however, able to make an OpgH depletion strain. This strain has a deletion of *opgH* at the native locus with a vanillate-inducible copy of *opgH* at the *vanA* locus (EG3421). As we do not have an antibody for OpgH, we generated a second depletion strain (EG3957) with OpgH-3×Flag produced under xylose-inducible control to assess the timing of depletion of the protein, under the assumption that the small tag would not significantly impact its depletion kinetics. When grown in the absence of vanillate, OpgH-3×Flag was depleted by 80% within 5 h and was completely undetectable at 24 h of depletion (Fig. S1A and B).

**Fig 1:**
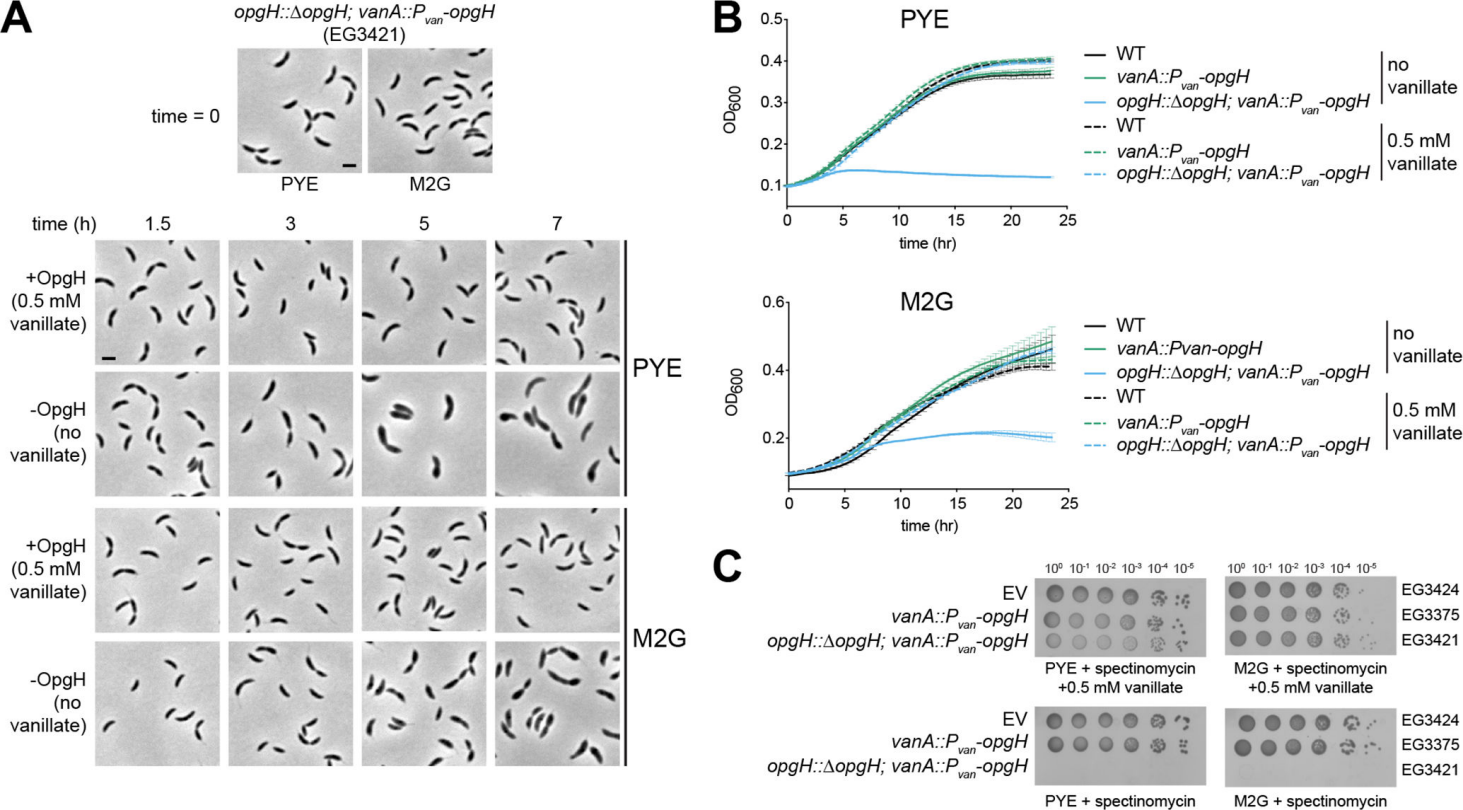
OpgH is essential for viability.(A) Phase contrast images of OpgH depletion strain (EG3421) in the presence or absence of 0.5 mM vanillate for 7 h. Cells were either grown in complex media (PYE) or minimal media (M2G) as indicated. Scale bar (2 µm) applies to every image in the panel. (B) Growth curve of WT (EG865), OpgH over-producing (EG3375), and OpgH depletion (EG3421) strains in PYE or M2G. Error bars represent ±1 SD of three biological replicates. (C) Spot dilutions of empty vector (EV; EG3424), OpgH over-producing (EG3375), and OpgH depletion (EG3421) strains on PYE or M2G plates with indicated antibiotics and inducer.

When grown with vanillate to induce *opgH* expression (+OpgH), cells looked morphologically wild type (WT) in both the complex medium peptone yeast extract (PYE) and in defined minimal medium (M2G) (Fig. 1A, *t* = 0). With OpgH, cells grew comparably to WT *Caulobacter* in both liquid (measured by optical density) (Fig. 1B) and solid media (measured by spot dilution) (Fig. 1C). When OpgH was depleted for 3 h in PYE without vanillate (−OpgH), cells exhibited a slight elongation and widening of the cell body. This phenotype was exacerbated during extended depletion, with cells showing prominent morphological defects at 5 and 7 h of depletion, such as asymmetric bulging (Fig. 1A). This bulging phenotype was also present for cells grown in M2G, but began later in the course of depletion, likely owing to the longer doubling time of *Caulobacter* in M2G (Fig. 1A). In addition to morphological defects, prolonged depletion of OpgH quickly became lethal in PYE or M2G, as seen by growth curve (Fig. 1B) and spot dilution (Fig. 1C) assays. Slight overexpression of *opgH* from the vanillate-inducible promoter in the presence of native *opgH* did not impact cell growth (Fig. 1B and C *vanA::P_van_-opgH*).

Prior work has demonstrated that OPGs are important for surviving low osmolarity environments; consequently, increasing the osmolarity of the media can improve the defects associated with OpgH deletion in other organisms (6, 17, 18). We therefore tested if high osmolarity media would support growth during OpgH depletion (Fig. S1C). When grown on solid media containing high salt (20 mM NaCl) or high sucrose (2.5% sucrose), the vanillate-inducible *opgH* strain grew comparably to WT in the presence of vanillate. On high osmolarity media lacking vanillate, however, cells depleted of OpgH failed to grow, indicating the importance of OpgH in *Caulobacter* even in high osmolarity conditions.

### OpgH depletion causes morphological defects including asymmetric bulging

**Fig 2:**
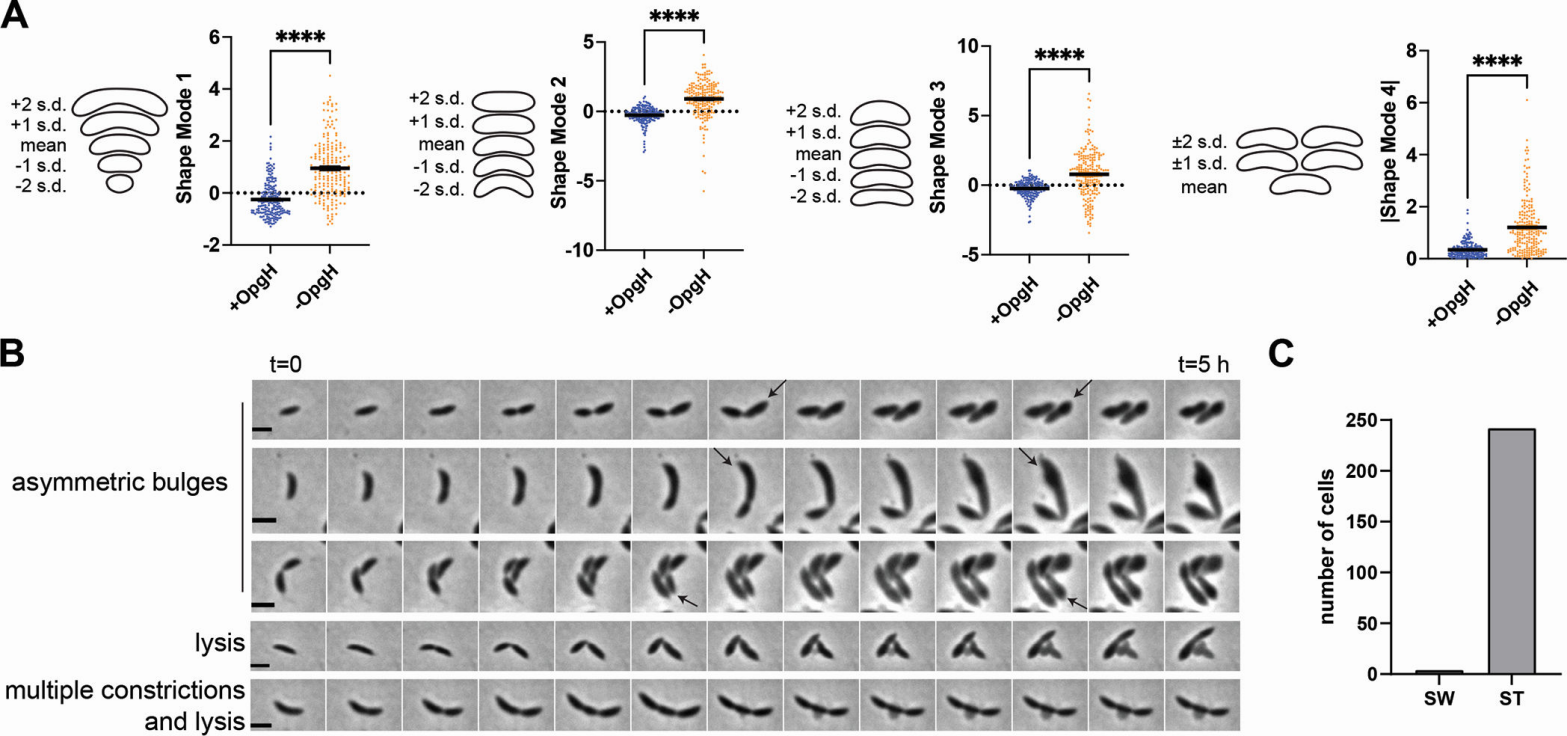
Cells lacking OpgH have morphological defects and bulging in the stalk-proximal region of the cell. (**A**) Principal component analysis (PCA) of the OpgH depletion strain (EG3421) after 5 h with 0.5 mM vanillate (blue, +OpgH) or without vanillate (orange, −OpgH). Scatter plots of 200 cells are shown for shape modes 1, 2, 3, and 4 which correspond to length, curvature, width, and asymmetric bulging. Contours are shown on the left to indicate the mean shape and 1 or 2 standard deviations from the mean for each shape mode. The absolute values are shown for shape mode 4. Statistical analysis uses a Mann-Whitney unpaired *t* test. ****=*P* < 0.0001. (**B**) Examples from time-lapse micrographs of prominent phenotypes of OpgH depletion for 5 h (EG3421). Black arrows indicate old (stalked) pole. Scale bar (2 µm) applies to every image in the panel. (**C**) Quantification of the number of cells exhibiting bulging in the swarmer (SW) or stalked (ST) pole from same time-lapse micrographs as in panel **B**. A total of 246 cells were counted. Bulging occurred in the stalked pole for 242 cells and in the swarmer pole for four cells.

The unique bulging phenotype of the depletion observed by eye motivated us to quantify the shape changes resulting from loss of OpgH. Using CellTool (19) to perform principal component analysis of cell morphology, we analyzed the OpgH depletion strain after five hours in the presence or absence of vanillate in PYE. We saw a statistically significant difference between cells with OpgH present (+OpgH) compared to cells without OpgH (−OpgH) in four shape modes (Fig. 2A). These shape modes approximately reflected the following features: shape mode 1, length; shape mode 2, curvature; shape mode 3, width; and shape mode 4, asymmetric bulging. Cells depleted of OpgH for 5 h were typically longer, less curved, and wider than cells with OpgH (Fig. 2A). For shape mode 4, which reflects asymmetric bulging, we have reported the absolute values, since the bulging can appear on either side of a cell outline. Our analysis indicates that cells producing OpgH rarely, if ever, exhibit asymmetric bulging while this is frequently observed in the OpgH-depleted condition (Fig. 2A). We also measured the shape variance of the four shape modes when OpgH was depleted in M2G. The OpgH-depleted cells in M2G (M2G − OpgH) have statistically significant differences in shape modes 1 through 4 compared to cells with OpgH present (M2G + OpgH) (Fig. S2).

We were especially interested in the unique asymmetric bulging phenotype of OpgH-depleted cells. To investigate this asymmetry, we leveraged the inherent cell polarity of *Caulobacter* to determine if the bulging consistently occurred at the same pole or was random. Every *Caulobacter* cell has a defined orientation, with a stalked pole and a swarmer pole, that can be visually tracked over the course of a few cell cycles. We performed time-lapse microscopy of the OpgH-depleted cells in the absence of vanillate to observe depletion for 5 h. The most notable phenotypes are summarized in Fig. 2B, including asymmetric bulging, elongation, multiple constriction events, and lysis. From our time-lapse analysis, we were able to follow single cells through complete cell cycles, which allowed us to orient the cells and identify the stalked (old) or swarmer (new) pole of a given cell. As illustrated in the top three examples in Fig. 2B (asymmetric bulges), we determined that nearly all bulging (~98%) occurs in the stalked half of the cell (nearest to the old pole, indicated by black arrows) (Fig. 2B and C). We localized a mNeonGreen fusion to OpgH (OpgH-mNG) and found that it was diffuse along the entire body of the cell in both PYE and M2G media (Fig. S3). These observations suggest that bulging is not related to OpgH localization, but to differential sensitivity of one or more factors unique to the stalked half of the cell during OpgH depletion.

### The predicted structures of *Caulobacter* and *E. coli* OpgH suggest similarities and differences between OpgH in these species

OpgH has been best studied in *E. coli*, where it has been genetically and biochemically implicated in the production of OPGs using UDP-glucose as a substrate (20, 21). *E. coli* OpgH (EcOpgH) has an auxiliary role, as well, acting as a direct inhibitor of FtsZ that coordinates cell size with nutrient availability (16). Deletion of *opgH* results in a mild reduction in cell length and overproduction of OpgH causes filamentation. The enzymatic activity of EcOpgH is not required to regulate FtsZ, and the FtsZ regulatory function has instead been attributed to the N-terminal region of EcOpgH. Since we observe morphological defects with depletion of *Caulobacter* OpgH (CcOpgH), we sought to compare the sequences and predicted structures of EcOpgH and CcOpgH to understand if CcOpgH may perform a similar moonlighting function to EcOpgH.

The Alphafold Protein Structure Database includes predicted structures for both EcOpgH (AF-P62517-F1) and CcOpgH (AF-B8GX72-F1), with very high model confidence scores for the majority of residues in each (22, 23). We aligned the primary sequences of EcOpgH and CcOpgH (Fig. S4A), as well as the predicted structures of each (Fig. S4B through D). The aligned structures have an overall RMSD of 0.678, indicating high structural similarity (Fig. S4D). The structures align particularly well in the predicted transmembrane regions and in the adjacent cytoplasmic domain predicted to be required for binding UDP-glucose, which is consistent with the conservation of enzymatic function. However, as is clear from both the sequence and structural alignments, EcOpgH bears extensions in both the N-terminal (gray) and C-terminal (salmon) regions compared to CcOpgH that fold into an extension of the cytoplasmic domain that is absent in CcOpgH (Fig. S4A and C). Moreover, there is little to no sequence conservation between the N- or C-terminal regions of CcOpgH and those of EcOpgH. Since FtsZ inhibition is attributable to the N-terminal region of EcOpgH, this, along with our observation that CcOpgH does not localize to midcell in *Caulobacter* cells (Fig. S3) and the distinct phenotypes observed with loss of OpgH between *E. coli* and *Caulobacter*, all suggests that CcOpgH likely does not regulate FtsZ in the same way as EcOpgH. We also note extensions in the predicted periplasmic loops of EcOpgH that are absent in CcOpgH. These could be sites where EcOpgH interfaces with periplasmic partners like OpgG, which is not encoded in *Caulobacter*.

### Glycosyltransferase activity of OpgH is required to maintain proper morphology

**Fig 3:**
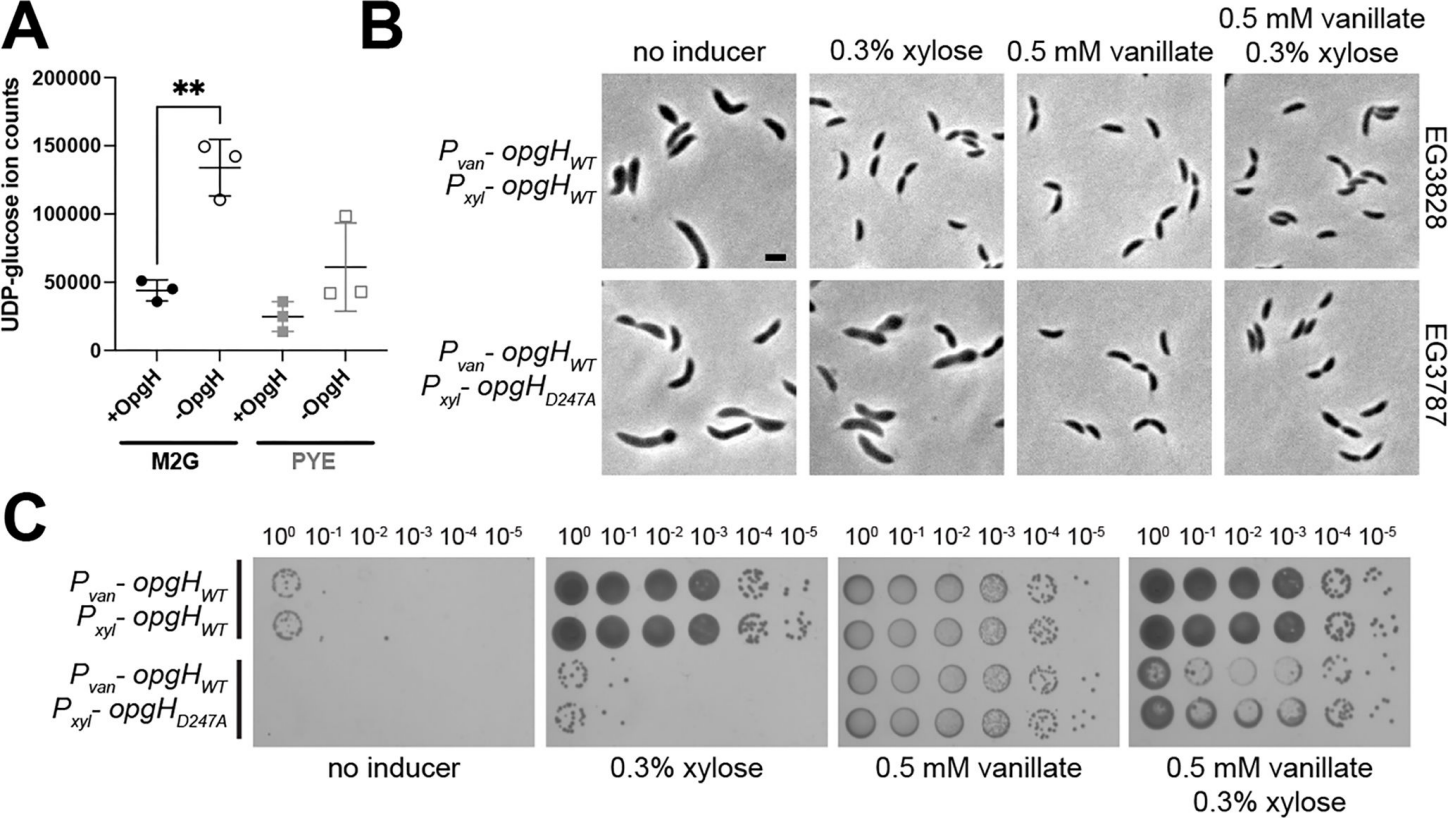
OpgH enzymatic activity is essential for morphogenesis. (**A**) Levels of UDP-glucose in extracts from cells producing OpgH (+OpgH) or depleted of OpgH for 5 h (−OpgH) in the indicated media. Mean and standard deviation are plotted. **, *P* = 0.0022 using unpaired *t* test. (**B**) Phase contrast images of the OpgH depletion strain (∆*opgH* + P_van_ *opgH*) with xylose inducible WT *opgH* (EG3828) or *opgH_D247A_* (EG3787). Cells have been depleted or induced with the indicated additive for 5 h. Scale bar (2 µm) applies to every image in the panel. (**C**) Spot dilutions of the same strains grown on PYE with indicated inducer for 2 days. Two biological replicates of each are presented.

Given that OpgH is characterized as an OPG synthase in *E. coli* and the similarity between the predicted structures of *E. coli* and *Caulobacter* OpgH, we wondered if it has a similar enzymatic function in *Caulobacter*. We attempted to demonstrate a role for *Caulobacter* OpgH in producing OPGs using established methods for isolation and detection of *E. coli* OPGs (11). However, we were unable to detect OPGs using these methods, perhaps reflecting the unique chemistry of *Caulobacter* OPGs compared to *E. coli* OPGs (9). Instead of assessing the effects of OpgH on its putative product, we sought to test if depletion of OpgH altered cellular levels of its predicted substrate, UDP-glucose. To this end, we extracted and quantified polar metabolites from cells producing OpgH or cells depleted of OpgH for 5 h in either defined (M2G) or complex (PYE) media. We found that levels of UDP-glucose increased two- to threefold when OpgH was depleted in either media condition (Fig. 3A; Table S1), consistent with the hypothesis that *Caulobacter* OpgH converts UDP-glucose to an OPG product.

In *E. coli*, the enzymatic activity of OpgH is dispensable for viability and for its effects on cell division. To probe the importance of the enzymatic activity of *Caulobacter* OpgH in promoting morphogenesis, we sought to characterize the role of predicted active site residues. Enzymes in the GT-A family of glycosyltransferases, like OpgH, all contain a conserved D-X-D motif (amino acids 245–247 [D-A-D] in *Caulobacter* OpgH) (Fig. S4A), which are responsible for binding the phosphate group on a nucleotide donor and coordinating a divalent cation required for activity (24). Mutation of either aspartate residue in the D-X-D motif eliminates the glycosyltransferase activity *in vitro* of other enzymes within this family, while not disrupting the fold of the enzyme (25).

We attempted to convert the latter aspartate in the *Caulobacter* OpgH D-X-D motif to an alanine (OpgH_D247A_) at the native *opgH* locus, but were unsuccessful. This initial observation suggested that the enzymatic activity of OpgH may be essential. We therefore investigated the phenotype of cells producing this variant protein by creating a strain harboring a native deletion of *opgH*, along with a vanillate-inducible copy of WT OpgH and a xylose-inducible copy of either WT *opgH* or the *opgH_D247A_* variant. Depletion of both copies of OpgH resulted in the expected elongation and bulging phenotype, and expression of WT *opgH* yielded normal growth and morphology. However, production of OpgH_D247A_ phenocopied depletion of OpgH (Fig. 3B). We verified the lethality of the OpgH_D247A_ mutant with spot dilutions (Fig. 3C). To ensure that the D247A mutation did not destabilize OpgH, we assessed the function and production of 3×Flag-tagged WT or OpgH_D247A_ during depletion of OpgH. Consistent with the untagged variants, WT OpgH-3×Flag supported growth while OpgH_D247A_-3×Flag did not, despite each protein being stably produced as confirmed by immunoblotting (Fig. S5). The identification of a catalytically dead mutant of OpgH that phenocopies its depletion implicates OpgH’s glycosyltransferase activity in maintaining cellular morphology.

### Cell wall synthesis is disrupted during OpgH depletion

**Fig 4:**
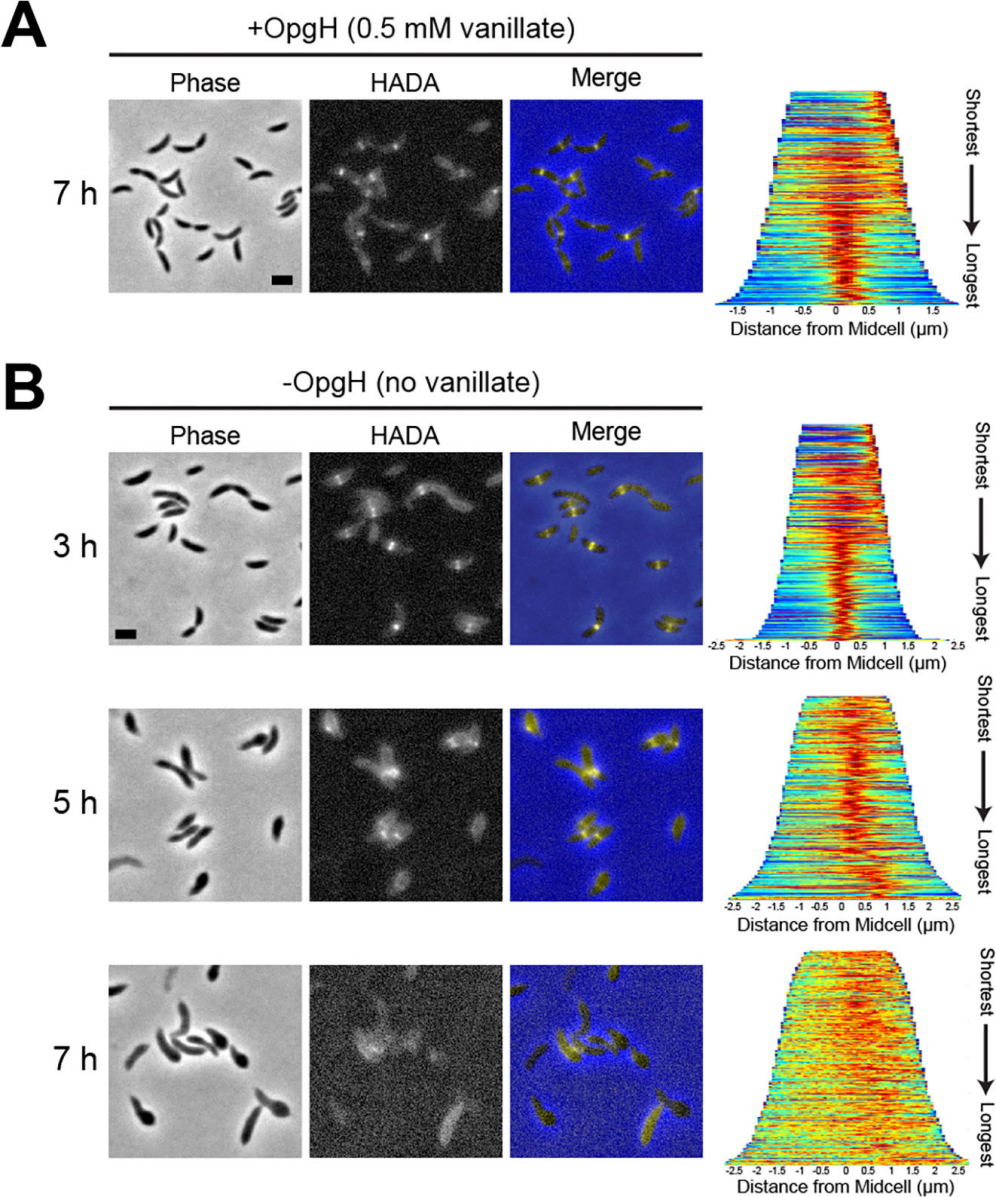
OpgH depletion results in misincorporation of cell wall material. Phase contrast, epifluorescence, and merged images showing HADA incorporation (**A**) with vanillate at 7 h and (**B**) without vanillate for 7 h in the OpgH depletion strain (EG3421). Demographs (right) show the normalized HADA intensities across the population, arranged from shortest to longest cell for 300 cells. Scale bar (2 µm) applies to every image in the panel.

The primary determinant of cell shape is the PG cell wall, so we hypothesized that bulging is a result of misregulated PG synthesis. We therefore sought to visualize active PG synthesis in OpgH-depleted cells. Using the fluorescent D-amino acid, HADA (26), we captured a snapshot of active PG synthesis over the course of OpgH depletion. The HADA patterning of the OpgH depletion strain grown with vanillate (+OpgH) corresponded with the expected localization of PG synthesis in WT *Caulobacter*. HADA incorporated at the cell pole and/or broadly along the cell body in swarmer cells and then localized to midcell in stalked and pre-divisional cells (Fig. 4A). When vanillate was removed and OpgH was depleted (−OpgH), we began to observe atypical HADA incorporation starting at 5 h (Fig. 4B). At this time point, HADA incorporation was more diffuse but still yielded a thick band near midcell that was typically adjacent to the asymmetric bulges and closer to the new cell pole than the old pole (Fig. 4B). By 7 h of depletion, HADA incorporation was almost entirely diffuse and, in many cases, we observed minimal incorporation (Fig. 4B). From these data, we conclude that PG synthesis is spatially perturbed in the absence of OpgH.

### The divisome and elongasome are mislocalized in OpgH-depleted cells

**Fig 5:**
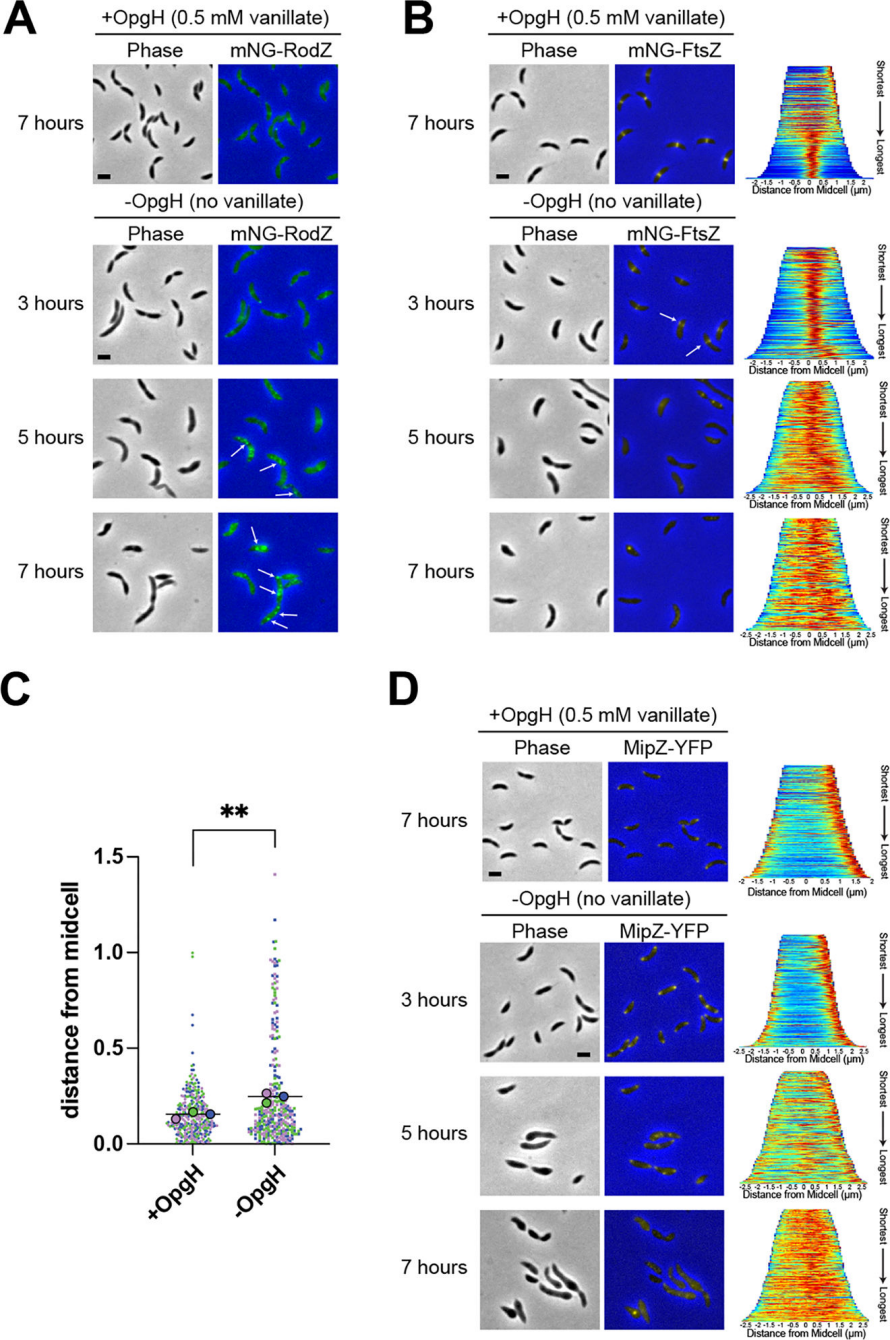
Divisome and elongasome proteins are mislocalized in OpgH-depleted cells. Phase contrast and epifluorescence merged images of (**A**) mNG-RodZ (EG3790), (**B**) mNG-FtsZ (EG3770), and (**D**) MipZ-YFP (EG3808) localization of OpgH depletion strains with (at 7 h) and without 0.5 mM vanillate (at 3, 5, and 7 h). White arrows indicate examples of (**A**) mNG-RodZ puncta and (**B**) asymmetric mNG-FtsZ localization. Demographs for FtsZ and MipZ localizations present the normalized intensities for cells across the population, arranged from shortest to longest (right). (**C**) Distance of the Z-ring from midcell (µm) in the presence of vanillate (+OpgH) or after OpgH depletion for 3 h (no vanillate, −OpgH). Colors indicate measurements from three biological replicates, each of which has 100 cells. Small symbols reflect individual cell measurements, large symbols indicate mean for each replicate. Line indicates overall mean. **, *P* = 0.0074 between the means of the three replicate means using unpaired *t* test. Scale bar (2 µm) applies to every image in the panel.

The changes in PG synthesis we observed during OpgH depletion align with the profound morphological defects that occur without OpgH. We hypothesized that misregulation of PG synthesis could be attributed to mislocalization of the PG synthetic machinery associated with the divisome or elongasome. We began by characterizing the localization of mNeonGreen-RodZ (mNG-RodZ) under the control of its native promoter. RodZ is an essential part of the elongasome and its localization corresponds to sites of PG synthesis (27). RodZ localization is dependent on MreB, the actin homolog that directs localization of elongasome proteins (27). In WT cells, RodZ and MreB exhibit patchy localization that focuses at midcell in stalked and predivisional cells (27, 28). We observed this expected localization pattern for mNG-RodZ in our OpgH depletion strain in the presence of vanillate (+OpgH) (Fig. 5A). In contrast, when OpgH was depleted, RodZ formed intense puncta by 5 h of depletion, which was exacerbated by 7 h (Fig. 5A). Additionally, we noticed an increase in mNG-RodZ signal over the course of depletion (Fig. S6A). Since *mNG-rodZ* expression was under the control of the native *rodZ* promoter, this could be attributed to an inability to turn over RodZ or a mechanism to increase the production of RodZ as OpgH is depleted.

Since RodZ’s midcell localization is also dependent on FtsZ, we next assessed FtsZ localization in the OpgH depletion strain. FtsZ is the essential master regulator of cell division, forming a dynamic scaffold that directs assembly of the division machinery to the future division site (29). When OpgH was present, mNG-FtsZ consistently formed tight midcell bands in stalked and predivisional cells (Fig. 5B, +OpgH). In contrast, when OpgH was depleted, mNG-FtsZ formed slightly diffuse bands by 3 h of depletion that appeared to be shifted away from midcell (Fig. 5B, −OpgH, white arrows). This off-center FtsZ localization potentially aligns with the asymmetric HADA incorporation (Fig. 4B) and bulging we observed. We determined the position of Z-rings in cells producing OpgH or cells depleted of OpgH for 3 h and found that Z-rings were, indeed, positioned more asymmetrically (i.e., further from midcell) during OpgH depletion than in the presence of OpgH (Fig. 5C). By 5 h of depletion, FtsZ was almost entirely diffuse (Fig. 5B). This aberrant Z-ring placement explains the inability of RodZ to properly localize, and suggests that, by 5 h of OpgH depletion, both the divisome and elongasome are unable to properly localize and direct PG synthesis.

We next turned our attention to the Z-ring positioning protein, MipZ, which is a negative regulator of FtsZ assembly (30). MipZ forms a unipolar focus in swarmer cells, then becomes bipolar as the origin of replication is segregated. Bipolar MipZ inhibits FtsZ polymerization at the poles and directs Z-ring formation at midcell. MipZ-YFP localization in cells with OpgH was consistent with the previously reported unipolar to bipolar MipZ localization over the *Caulobacter* cell cycle (Fig. 5D, +OpgH). After 5 h of OpgH depletion, however, MipZ-YFP localization was largely perturbed, with more diffuse MipZ and with some cells exhibiting three or more MipZ foci (Fig. 5D, −OpgH). After 7 h of OpgH depletion, MipZ-YFP was almost entirely diffuse, while only sometimes forming puncta (Fig. 5D). We propose that mislocalization of MipZ in cells lacking OpgH is a key factor that leads to mislocalization of the divisome and elongasome, yielding the pleiotropic morphological defects we have observed.

### Overexpression of *cenR* phenocopies depletion of OpgH

**Fig 6:**
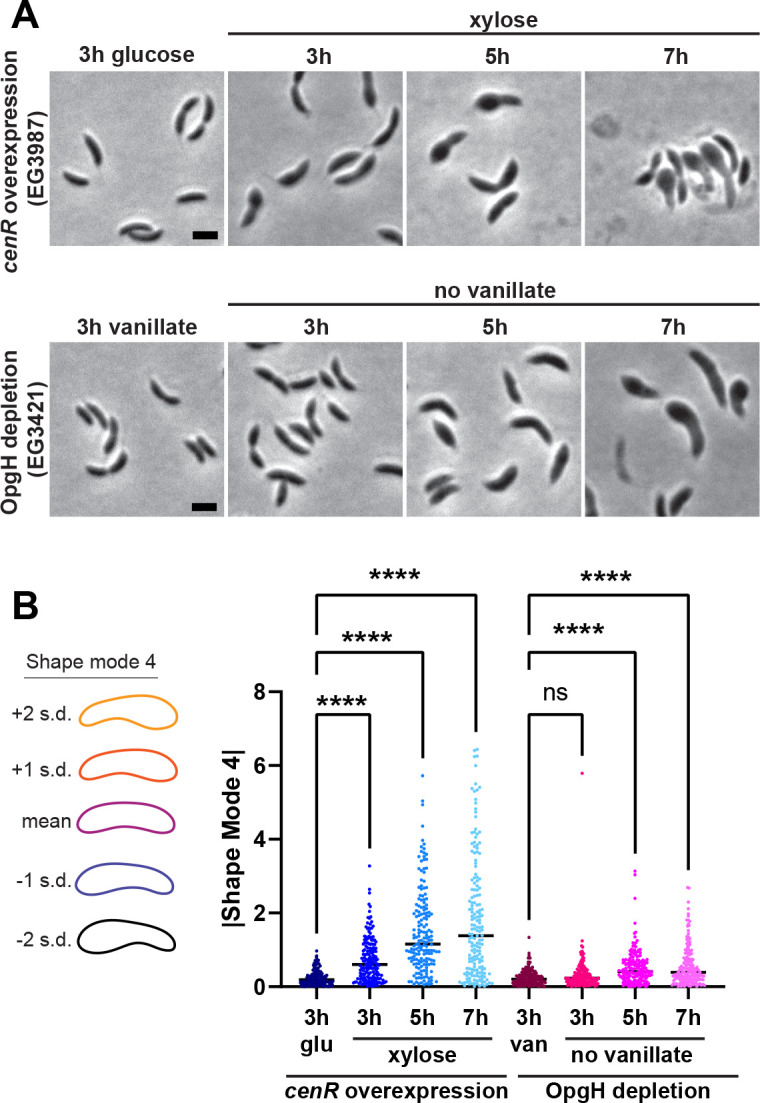
Overexpression of *cenR* phenocopies OpgH depletion. (**A**) Phase contrast images of *cenR* overexpression (EG3987) in the presence of 0.3% xylose and OpgH depletion (EG3421) in the absence of 0.5 mM vanillate for 7 h. Cells were grown in complex media (PYE). Scale bar (2 µm) applies to every image in the panel. (**B**) Principal component analysis (PCA) for shape mode 4 (asymmetric bulging) of the *cenR* overexpression strain (EG3987) and the OpgH depletion strain (EG3421). Cells were grown for 3, 5, and 7 h in PYE with 0.3% xylose for *cenR* overexpression or lacking vanillate to deplete OpgH. Uninduced *cenR* (3 h glu) and induced *opgH* (3 h van) serve as controls. Scatter plots of 173 cells are presented. Contours indicate the mean shape and 1 or 2 standard deviations from the mean. Shape mode 4 is plotted as the absolute value. Statistical analysis uses a Mann-Whitney unpaired *t* test. ****, *P* < 0.0001.

We have found that OpgH depletion perturbs the localization of divisome and elongasome components, which ultimately disrupts PG synthesis. These observations caused us to hypothesize that depletion of OpgH compromises the integrity of the cell envelope and could trigger a stress response. In *Caulobacter* and the related α-proteobacterium *Rhodobacter sphaeroides*, the CenKR (Cell Envelope Kinase and Regulator) two-component system has been implicated in maintaining cell envelope homeostasis (15, 31, 32). Both *cenK* and *cenR* are essential in *Caulobacter*, and depletion of either prevents growth and results in membrane blebbing and cell lysis. Intriguingly, overactivation of the pathway via xylose-inducible expression of *cenR* looks phenotypically similar to OpgH depletion, including bulging that occurs only at the stalked pole (15). Indeed, direct comparison of OpgH depletion to overproduction of Flag-tagged CenR showed the characteristic bulging phenotype in both conditions (Fig. 6A).

These observations prompted us to use CellTool again, this time to analyze how asymmetric bulging (shape mode 4) changes over 3, 5, and 7 h of either *flag-cenR* overexpression or OpgH depletion (Fig. 6B). Relative to the uninduced sample (3 h glu), *flag-cenR* overexpression caused a statistically significant difference in asymmetric bulging at all time points, with each time point showing an increase in bulging compared to the one prior. We noted a similar trend for OpgH depletion relative to cells producing OpgH (3 h van); however, significant differences in asymmetric bulging were not detected until 5 h, which is consistent with our observations from the phase contrast imaging (Fig. 6A). These observations demonstrate that *cenR* overexpression and OpgH depletion result in the similar and unusual asymmetric bulging phenotype.

### Divisome and elongasome components are mislocalized during *cenR* overexpression

**Fig 7:**
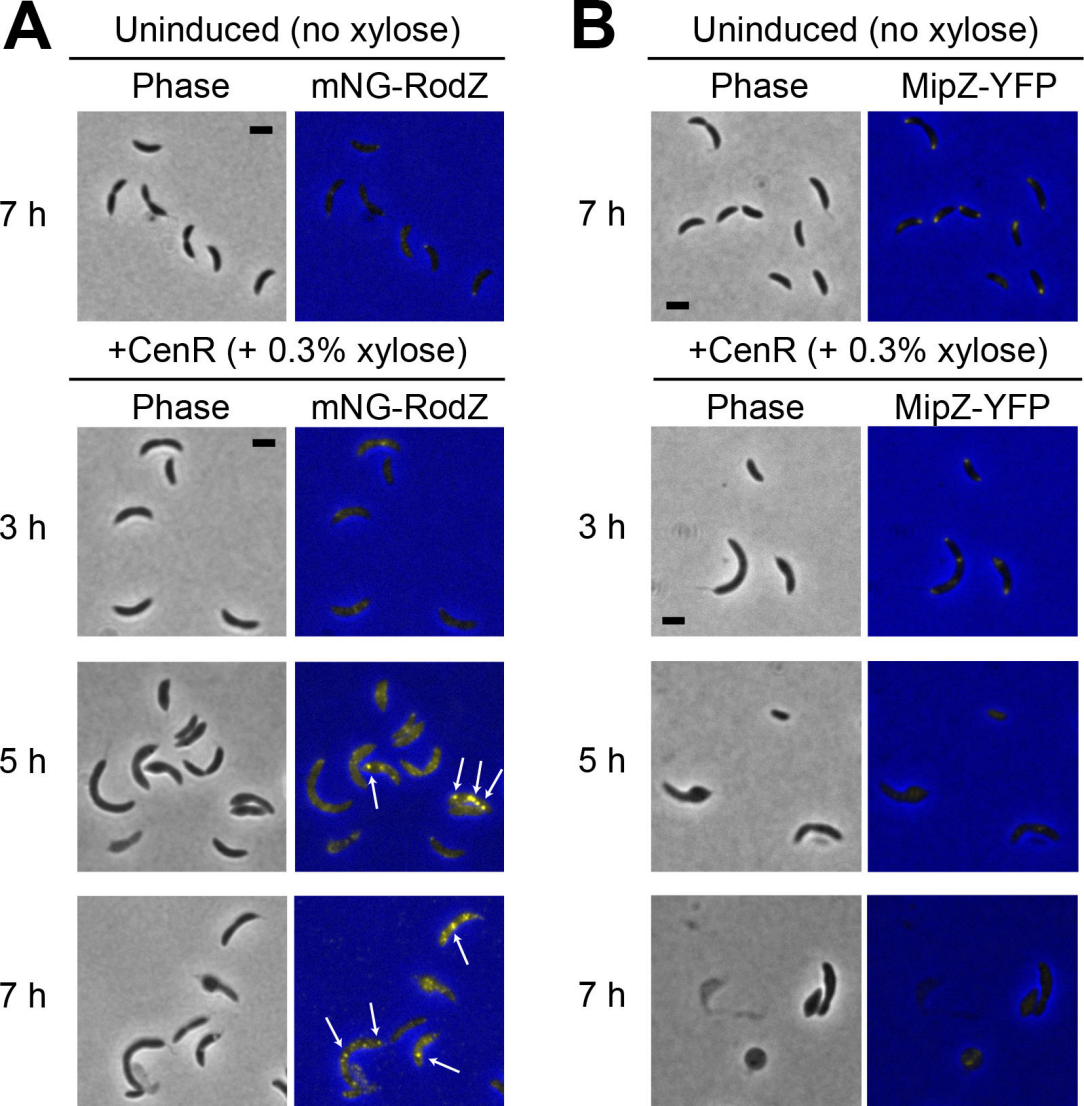
Divisome and elongasome proteins are mislocalized during *cenR* overexpression. Phase contrast and epifluorescence merged images of (**A**) mNG-RodZ (EG3990) and (**B**) MipZ-YFP (EG3989) localization of *flag-cenR* overexpression strains without (at 7 h) and with 0.3% xylose (at 3, 5, and 7 h). White arrows indicate examples of mNG-RodZ puncta. Scale bar (2 µm) applies to every image in the panel.

The distinctive phenotypic similarities between *cenR* overexpression and OpgH depletion caused us to further explore their relationship. Since we found mislocalization of RodZ and MipZ during OpgH depletion, we next sought to characterize their localizations during *flag-cenR* overexpression. We first assessed the localization of mNG-RodZ when expressed from its native promoter (Fig. 7A). Similar to what we observed for OpgH depletion, mNG-RodZ signal became diffuse and created bright foci throughout the length of the cell over the 7 h of xylose-induced *flag-cenR* overexpression (Fig. 7A; Fig. S6B).

We next localized MipZ-YFP. As with OpgH depletion, MipZ-YFP went from exhibiting a unipolar or bipolar localization when *flag-cenR* was not induced to having a diffuse localization during *flag-cenR* overexpression (Fig. 7B). There was also an overall decrease in MipZ-YFP signal at 5 and 7 h, which we did not observe during OpgH depletion. This may be attributed to the different methods of *mipZ-yfp* expression, while MipZ-YFP was produced from its native promoter during *cenR* overexpression, MipZ-YFP was produced from a xylose-inducible promoter during OpgH depletion. Nonetheless, both mNG-RodZ and MipZ-YFP localizations were perturbed during *flag-cenR* overexpression in a manner akin to what was observed during OpgH depletion.

### Levels of divisome and elongasome proteins are affected by OpgH depletion or *cenR* overexpression

**Fig 8:**
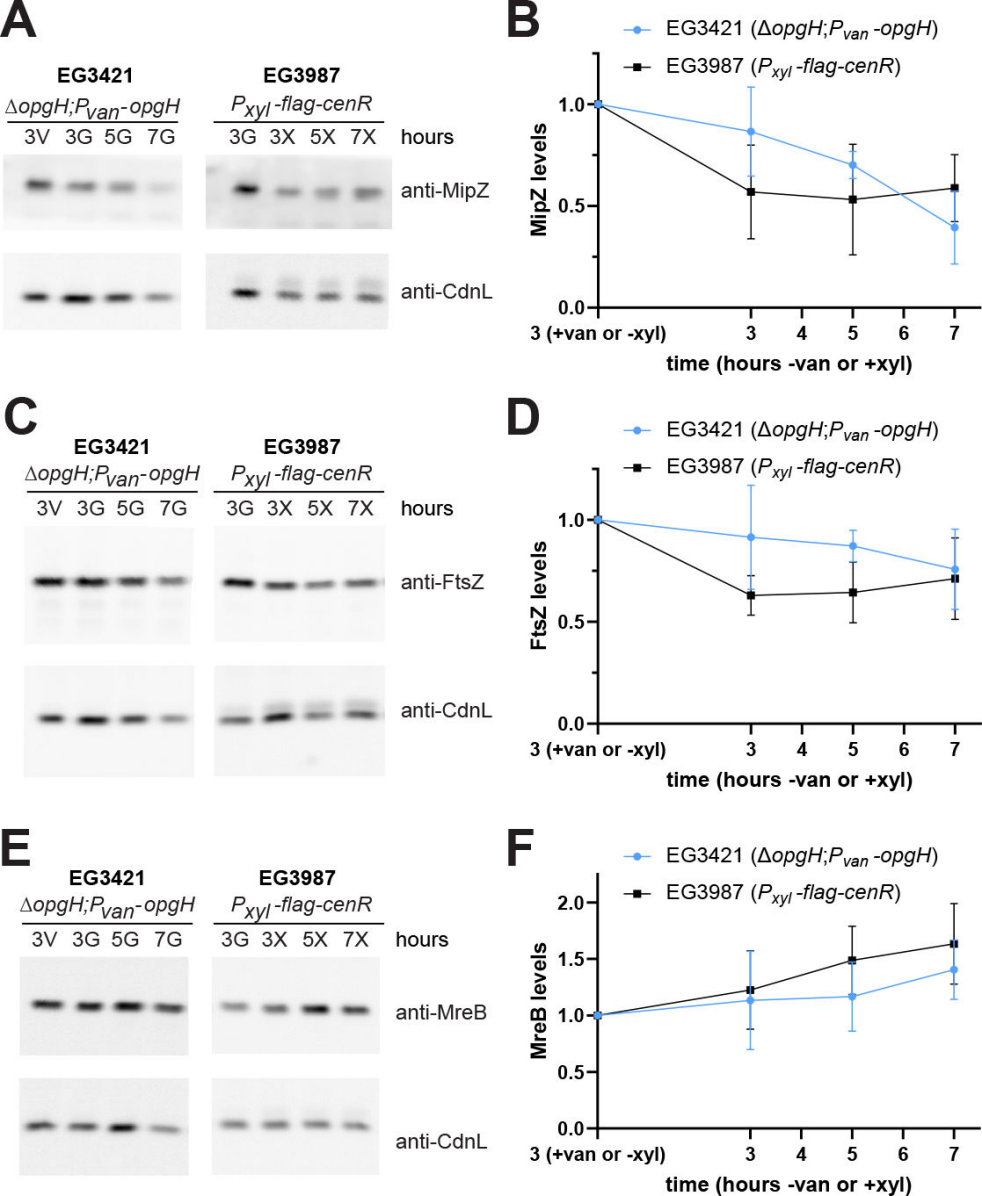
Divisome and elongasome protein levels are affected during OpgH depletion or *cenR* overexpression. Representative immunoblots and corresponding densitometry analysis for protein levels of (**A and B**) MipZ, (**C and D**) FtsZ, and (**E and F**) MreB in OpgH depletion (EG3421) and *flag-cenR* overexpression (EG3987) strains. CdnL was used as a loading control. Levels were normalized to total protein and plotted relative to 3 h of induced *opgH* (3 +van) or uninduced *flag-cenR* (3 –xyl). Error bars indicate ±1 standard deviation for three biological replicates.

The decrease in MipZ-YFP signal and increase in mNG-RodZ signal during *flag-cenR* overexpression prompted us to investigate how the levels of divisome and elongasome proteins are affected by *flag-cenR* overexpression or OpgH depletion. To this end, we sampled cell lysates during *cenR* overexpression or OpgH depletion at 3, 5, and 7 h, and then probed for MipZ, FtsZ, and MreB by immunoblotting. We first focused on the divisome. Consistent with what we observed for MipZ-YFP signal during *flag-cenR* overexpression, MipZ levels were reduced by about 50% after 3 h and then remained fairly constant through 7 h (Fig. 8A and B). During OpgH depletion, MipZ levels were close to that of OpgH-producing cells after 3 h but dropped as the depletion progressed, again demonstrating that the consequences associated with OpgH depletion are delayed relative to *cenR* overexpression. FtsZ protein levels followed similar trends to those observed for MipZ during both *flag-cenR* overexpression and OpgH depletion (Fig. 8C and D).

We next turned our attention to the elongasome and monitored MreB levels, as we were unable to find reliable antibodies for the detection of RodZ or mNG-RodZ. Interestingly, for both *flag-cenR* overexpression and OpgH depletion, MreB levels increased over the 7 h (Fig. 8E and F). This is similar to what we noted by eye for mNG-RodZ fluorescence intensity (Fig. 5A and 7A). These observations suggest that OpgH depletion and *cenR* overexpression both lead to downregulation of divisome components and upregulation of elongasome components.

### Depletion of OpgH during *cenR* overexpression is responsible for cell bulging

Our observations that overactivation of CenKR results in similar yet faster-acting consequences to OpgH depletion suggests that CenKR lay downstream of OpgH. We hypothesized that depletion of OpgH triggers activation of this two-component system, thus leading to changes in the levels of morphogenetic proteins and ultimate bulging and lysis. We therefore expected that during *cenR* overexpression, OpgH levels should remain constant or increase as a compensatory mechanism. To address this, we assessed levels of a 3×Flag-tagged OpgH that is chromosomally integrated at the native locus. Contrary to our hypothesis, upon overexpression of *flag-cenR*, we found that OpgH-3×Flag levels decreased (Fig. S7).

**Fig 9:**
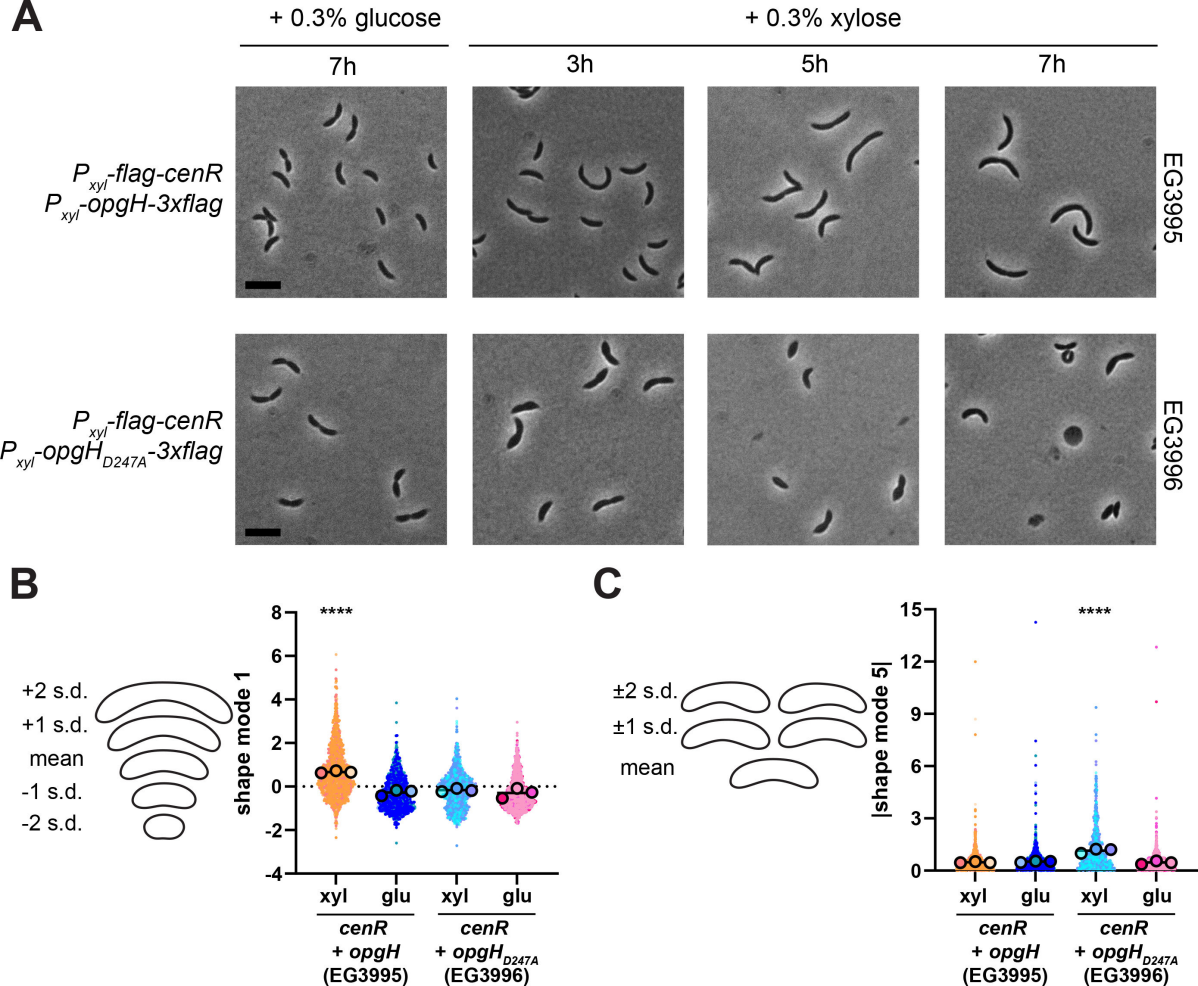
OpgH production during *cenR* overexpression prevents bulging. (**A**) Phase contrast images of *flag-cenR* and *opgH-3×flag* (EG3995) or *opgH_D247A_-3×flag* (EG3996) overexpression in the presence of 0.3% xylose (induced) or 0.3% glucose (uninduced control) for 7 h. Cells were grown in complex media (PYE). Scale bar (2 µm) applies to each image in the panel. (**B**) Principal component analysis (PCA) of the same strains after 7 h with 0.3% xylose (induced) or 0.3% glucose (uninduced) for shape mode 1 (length). Contours are shown on the left to indicate the mean shape and 1 or 2 standard deviations from the mean for each shape mode. (**C**) Same as panel **B** but for shape mode 5 (asymmetric bulging). The absolute values for contour standard deviations are shown. For both panels **B** and **C**, colors indicate measurements from three biological replicates. For the three EG3995 +xyl replicates, 535, 453, and 468 cells were used. For EG3995 +glu, 294, 466, and 466 cells were used. For EG3996 +xyl, 347, 317, and 161 cells were used. For EG3996 +glu, 689, 307, and 517 cells were used. Small symbols reflect individual cell measurements. Large symbols indicate mean for each replicate. Line indicates overall mean. Statistical analysis uses ANOVA (Dunnett’s multiple comparisons test) comparing each mean to the mean of EG3995 +glu. ****, *P* < 0.0001.

These findings raise another possibility: instead of overexpression of *cenR* causing bulging and lysis, this phenotype may be solely due to reduction in OpgH levels. To disentangle these scenarios, we constructed a strain for xylose-inducible co-expression of *flag-cenR* with *opgH-3×flag*. This strain allows us to assess the phenotypic consequences of *cenR* overexpression without downregulation of *opgH*. We compared this to a strain co-expressing *flag-cenR* with *opgH_D247A_-3×flag*, wherein OpgH protein is made but is not active for OPG synthesis. As expected, co-expression of *flag-cenR* with *opgH_D247A_-3×flag* resulted in bulging and lysis on a similar timescale to *flag-cenR* overexpression alone (Fig. 9A), which is consistent with our earlier observations that OpgH_D247A_ is catalytically inactive and unable to support proper morphology. Interestingly, expression of *opgH-3×flag* during *flag-cenR* overexpression prevented the bulging phenotype; however, cells became elongated and appeared to stop dividing (Fig. 9A). We again used CellTool to further analyze these phenotypes. Seven hours of overexpression of *flag-cenR* with *opgH-3×flag* resulted in cells that were significantly longer than cells incubated with glucose or cells expressing *flag-cenR* with *opgH_D247A_-3×flag* (Fig. 9B; Fig. S8). We confirmed that these cells did not bulge, as only cells overexpressing *flag-cenR* with *opgH_D247A_-3×flag* showed significant differences in that shape mode (Fig. 9C; Fig. S8).

**Fig 10:**
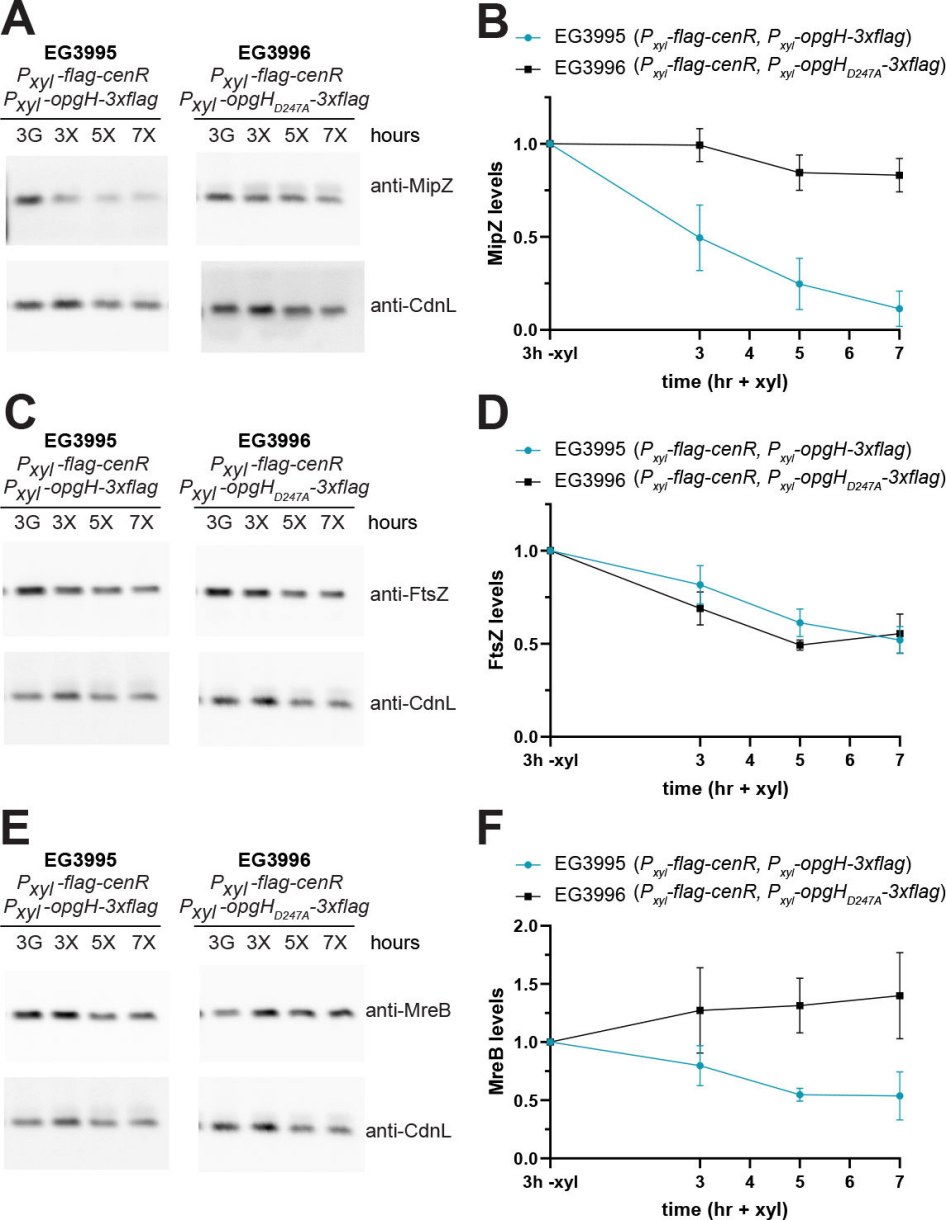
Divisome and elongasome protein levels are affected during *cenR* overexpression in the presence of OpgH. Representative immunoblots and corresponding densitometry analysis for protein levels of (**A and B**) MipZ, (**C and D**) FtsZ, and (**E and F**) MreB in *flag-cenR* and *opgH-3×flag* (EG3995) or *opgH_D247A_-3×flag* (EG3996) overexpression strains. CdnL was used as a loading control. Levels were normalized to total protein and plotted relative to 3 h uninduced (3 h − xyl). Error bars indicate ±1 standard deviation for three biological replicates.

**Fig 11:**
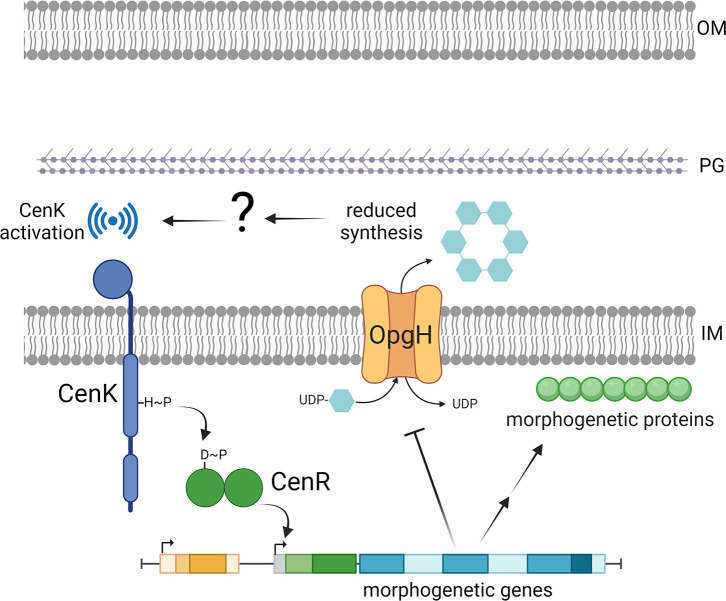
Model of OpgH- and CenKR-mediated regulation of morphogenesis. A reduction in OPGs via OpgH depletion leads to changes in envelope structure and/or periplasmic volume. By some currently unknown mechanism, these changes in OPG abundance lead to activation and autophosphorylation of the CenK sensor histidine kinase. CenK phosphorylates and activates the CenR response regulator, which impacts the transcription of morphogenetic genes by downregulating MipZ and FtsZ and upregulating MreB, leading to a reduction in cell wall synthesis. This would slow growth and division to allow for cell envelope repair. CenKR activation also leads to downregulation of OpgH. While this regulatory circuit is not fully understood, it is thought these components act together to maintain cell envelope integrity. OM = outer membrane; PG = peptidoglycan cell wall; IM = inner membrane. Created with BioRender.com.

The elongation of cells overexpressing both *cenR* and *opgH* suggested that components of the elongasome and divisome were still being misregulated. Indeed, similar to what we found during overexpression of only *flag-cenR*, induction of both *flag-cenR* and *opgH-3×flag* resulted in decreased MipZ and FtsZ levels (Fig. 10A through D compared to Fig. 8A through D). This indicates that CenR activation is responsible for downregulation of MipZ and FtsZ. Interestingly, MreB levels also decreased, which is opposite to what we found for induction of both *flag-cenR* and *opgH_D247A_-3×flag* or *flag-cenR* overexpression alone (Fig. 10E and F compared to Fig. 8E and F). Taken together, these observations suggest that downregulation of OpgH causes the bulging and lysis phenotype seen during OpgH depletion or *cenR* overexpression. However, downregulation of the divisome components results from activation of CenKR, suggesting a regulatory circuit between OpgH depletion and CenKR activation: OpgH depletion triggers CenKR activation, thus affecting levels of morphogenetic proteins as well as negatively regulating OpgH, leading to bulging (Fig. 11).

## DISCUSSION

In this work, we have highlighted the essential and previously undiscovered role of OpgH in *Caulobacter* morphogenesis, and we have connected OpgH to the CenKR two-component system. We have demonstrated that *opgH* is essential (Fig. 1) and characterized the unique morphological defects associated with loss of OpgH. Without OpgH, *Caulobacter* cells become elongated and develop asymmetric bulges. These bulges always occur in the stalk-proximal region of the cell, and result in lysis (Fig. 2). Interestingly, we can attribute the morphological defects of OpgH depletion to the glycosyltransferase activity of OpgH, as a catalytically dead OpgH mutant phenocopies the depletion (Fig. 3). Using PG labeling probes, we have shown that these phenotypes result from misregulation of PG insertion (Fig. 4), driven by disruption of the divisome and elongasome (Fig. 5). Strikingly, the morphology (Fig. 6) and morphogenetic protein localization and abundance (Fig. 7 and 8) associated with OpgH depletion phenocopy those of *cenR* overexpression. Ultimately, we determined that the bulging phenotype is due to the depletion of OpgH by *cenR* (Fig. 9). However, misregulation of the divisome and elongasome during OpgH depletion is caused by *cenR* activity (Fig. 10), suggesting a regulatory circuit between OpgH, CenKR, and the PG synthetic machinery (Fig. 11).

Our data implicate the glycosyltransferase activity of OpgH in the morphological defects associated with its loss. However, we do not yet have direct evidence that *Caulobacter* OpgH can synthesize OPGs. Indeed, although OpgH-dependent synthesis of OPGs has been observed in, for example, *E. coli* cell extracts, OpgH activity has not been reconstituted with purified protein *in vitro* from any organism (20). We recently identified a genetic interaction between OpgH and the novel OPG metabolic enzymes, EstG and BglX (9). In that study, we determined that the substrate of EstG is a cyclic hexamer of glucose, which is the first OPG identified in *Caulobacter* (9). Although our data support the hypothesis that OpgH converts UDP-glucose to OPGs, further biochemical studies are necessary to determine if this cyclic OPG is, in fact, the product of OpgH.

Our results also indicate a connection between OPG synthesis and maintenance of the levels and localization of divisome and elongasome components. This connection may be mediated by the CenKR two-component system, as we observed that the levels and localization of the divisome and elongasome change in a similar manner during OpgH depletion and *cenR* overexpression. The similarity in the phenotypes of OpgH depletion and *cenR* overexpression therefore leads us to believe that depletion of OpgH could compromise the integrity of the cell envelope, thus triggering CenKR and subsequently impacting the localization and levels of divisome and elongasome components (Fig. 11). Indeed, OPGs are thought to be important for maintaining proper osmolarity within the periplasm, thus impacting cell envelope structure (6), and CenKR has also been implicated in maintaining cell envelope homeostasis in both *Caulobacter* and the related α-proteobacterium *R. sphaeroides* (15, 31, 32). Similar to our findings, work in *R. sphaeroides* revealed misregulation of PG insertion and mislocalization of morphogenetic complexes when CenKR activity is increased (32). Additionally, *R. sphaeroides* CenKR has been shown to indirectly regulate the expression of peptidoglycan biosynthetic genes (31). While the CenKR regulon in *Caulobacter* is currently unknown, our observations suggest that CenKR regulates similar types of genes, thereby further connecting CenKR to OpgH and cell envelope homeostasis.

It is unclear what exactly CenK senses, however. While it is possible that CenK senses the concentration of another factor or molecule, such as OPGs, it is also possible that CenK measures periplasmic volume. In support of this, overexpression of *cenK* in *R. sphaeroides*, which increases CenKR activation in that organism, increases the average width of the periplasmic space (32). This suggests that CenKR plays a role in finetuning periplasmic volume. It has been proposed that a minimum periplasmic space is required for movement of PG synthetic machinery (33, 34). Therefore, it is plausible that when the periplasmic volume shrinks, as it does when OPG synthesis is downregulated (35), CenKR is activated to restrain cell division by modulating levels of morphogenetic proteins, thus allowing time for other mechanisms to increase the volume of the periplasm and provide room for PG synthesis to resume. A caveat to this model is that we found overexpression of *cenR* leads to reduced OpgH levels (Fig. 9A). However, by overexpressing *cenR* on a high-copy plasmid, we are pushing the activation of this pathway to the brink of cell death. Due to the essentiality of CenKR and OpgH in *Caulobacter*, there would almost never be a time in nature to reach this extreme activation state. Rather, these components are likely always working to continually sense the environment and regulate periplasmic width to control growth and division, and so the connection may be much more nuanced.

Of the phenotypes associated with OpgH depletion and *cenR* overexpression, we were most surprised by the asymmetric nature of the bulging phenotype. We have shown this phenotype is a consequence of OpgH depletion, as co-overproduction of both OpgH and CenR prevented bulging. Typically, cell bulging is a consequence of misregulation of either the elongasome/Rod complex or the divisome. Although *Caulobacter* cells are inherently polarized, the bulging that results from misregulation of the elongasome in *Caulobacter* (e.g., depleting MreB) (28) is uniformly distributed along the cell length, resulting in lemon-shaped cells. Bulging from a divisome mutant (i.e., a variant of FtsZ lacking its disordered linker [∆CTL]) (36, 37), results in envelope bulges only where ∆CTL is localized, that is, near midcell. The OpgH depletion phenotype is unique in its asymmetry, with bulging primarily on the stalk-proximal side of the cell. We observed that the Z-ring was more asymmetrically positioned after 3 h of OpgH depletion than in the presence of OpgH. We therefore propose that depletion of OpgH causes a shift in the location of the divisome and elongasome away from the stalked pole at early stages of OpgH depletion. This would deplete PG synthesis from the stalk-proximal region of the cell and, if accompanied by ongoing cell wall hydrolysis in that region, lead to local cell bulging. Alternatively, or in addition, it is possible that the asymmetry in bulging is due to an unidentified factor that directs PG synthesis or hydrolysis asymmetrically. For instance, loss of OpgH might lead to the misregulation of PG enzymes like l,d-transpeptidases, which primarily crosslink PG in the stalk (38, 39).

We have established that OpgH, and likely OPG production, plays a crucial role in *Caulobacter* morphogenesis and works in concert with CenKR to maintain the integrity of the cell envelope. The essentiality of *Caulobacter* OpgH and morphological defects associated with its loss provide an opportunity to elucidate the mechanism of action of OpgH and the OPG biosynthesis pathway. The previously studied OpgH homologs have all been nonessential, which limits the questions that we can ask. For instance, it is more challenging to study functional mutants *in vivo* or conduct genetic screens in an organism where OpgH is nonessential. Thus, this provides an appealing possibility for future work on *Caulobacter* OpgH, including avenues such as a mutagenesis screen to isolate novel mutants or a larger-scale functional domain analysis study. We have already identified functional OpgH mutants that suppress the lethality of cell envelope stresses in a hypersensitive mutant (9). These mutants, as well as extragenic mutations isolated from suppressor screens, will be valuable in elucidating the mechanistic role of OpgH and OPGs in cell envelope homeostasis. Our findings have established a fundamental homeostatic role for an essential OpgH homolog and have uncovered a novel connection between the OPG pathway, cellular morphology, and CenKR that is ripe for future investigation.

## MATERIALS AND METHODS

### *Caulobacter crescentus* growth media and conditions

*C. crescentus* NA1000 cells were grown at 30°C in peptone-yeast extract (PYE) medium. Xylose and glucose were used at a concentration of 0.3% (wt/vol) and vanillate at 0.5 mM for induction/depletion experiments. Prior to induction/depletion, cells were washed 3× in plain PYE or M2G and then resuspended in media containing the proper inducer or glucose. Antibiotics used in liquid (solid) medium as are follows: gentamycin, 1 (5) µg/mL; kanamycin, 5 (25) µg/mL; and spectinomycin, 25 (100) µg/mL. streptomycin was used at 5 µg/mL in solid medium. For growth curves, a Tecan Infinite M200 Pro plate reader measured absorbance every 30 min at OD_600_ of a 100-µL culture volume in a 96-well plate in biological triplicate with intermittent shaking. For spot dilutions, cells were grown to mid-log phase and diluted to an OD_600_ of 0.05. Cells were then serially diluted up to 10^−6^ and 5 µL of each dilution was spotted onto a PYE plate with indicated inducer and/or antibiotic. Plates were incubated at 30°C for 48 h. Strains and plasmids used in this study are listed in Table S2.

### Atypical strain construction

We could not make the following strains in low osmolarity PYE media, so they were constructed in M2G minimal media: EG3421 (*opgH::*∆*opgH; vanA::P_van_-opgH*), EG3770 (*opgH::*∆*opgH; vanA::P_van_-opgH*, *xylX::P_xyl_-mNG-FtsZ*), EG3790 (*opgH::*∆*opgH; vanA::P_van_-opgH*, *xylX::P_rodZ_-mNG-rodZ*), EG3808 (*opgH::*∆*opgH; vanA::P_van_-opgH*, *xylX::P_xyl_-mipZ-YFP*)*,* EG3828 (*opgH::*∆*opgH; vanA::P_van_-opgH; xylX::P_xyl_-opgH*), EG3787 (*opgH::*∆*opgH; vanA::P_van_-opgH; xylX::P_xyl_-opgH_D247A_*), EG3957 (*opgH::*∆*opgH; vanA::P_van_-opgH; xylX::P_xyl_-3×-Flag-opgH*), and EG3959 (*opgH::*∆*opgH; vanA::P_van_-opgH; xylX::P_xyl_-3×-Flag-opgH_D247A_*), EG3995 (pHXM-*cenR; xylX::opgH-3×flag*), and EG3996 (pHXM-*cenR; xylX::opgH_D247A_-3×flag*). For 500 mL of M2G plates, 465 mL of water and 7.5 g agar (1.5%) were autoclaved. Once cooled, 25 mL of 20× M2 salts, 500 µL of 500 mM MgSO_4_, 500 µL of 10 mM FeSO_4_, 10 mM EDTA (Sigma F-0518), and 0.3% glucose were added. Additional antibiotics or media supplements for selection were added at this time. After initial strain construction, all strains were able to be grown in either M2G or PYE liquid media with appropriate inducers.

### Microscopy

Cells in exponential phase were immobilized on 1% agarose pads and imaged using a Nikon Eclipse Ti inverted microscope. Images in Fig. 1 to 5; Fig. S1 and S3 were acquired with a Nikon Plan Fluor 100× (NA1.30) oil Ph3 objective and Photometrics CoolSNAP HQ^2^ cooled CCD camera. Images in Fig. 6 to 10 were acquired with an Apochromat Phase Contrast DM 100× (NA1.4) oil objective and Photometrics Prime BSI Express sCMOS camera. Images were processed using Adobe Photoshop. Levels were adjusted to the same range across samples in a given experiment. Cell shape analysis was performed using CellTool and demographs were generated using Oufti. Z-ring position analysis was performed in MicrobeJ (40) using the “line” feature. Three independent replicates were imaged and analyzed for Z-ring positioning, and data were presented in SuperPlots (41). Inducible fluorescent fusion proteins were induced for 1 h with 0.3% xylose before the indicated imaging time point. Mean fluorescence intensities for strains expressing mNG-RodZ were calculated using MicrobeJ (40).

### Metabolomics sample preparation and analysis

Metabolomic samples were prepared as described previously (42). Briefly, cells were grown to an OD_600_ of 0.3 in 4 mL and filtered through 0.22 µm nylon filters (Millipore GNWP04700). The cells were quenched by placing the filter upside down in a 60 mm dish containing 1.2 mL pre-chilled quenching solution (40:40:20 acetonitrile:methanol:H_2_O + 0.5% formic acid) and incubated for 15 min at −20°C. Cells were removed by pipetting the quenching solution over the filter, added to chilled bead beating tubes containing 50 mg of 0.1 mm glass beads, and neutralized with 100 µL 1.9M NH_4_HCO. Cells were lysed using a Qiagen Tissulyzer at 30 Hz for 5 min. Samples were spun at 4°C for 5 min at 16,000 × *g* and transferred to pre-chilled tubes to remove debris.

Metabolomics was performed at the Metabolomics Core Facility at Rutgers-Robert Wood Johnson Medical School. Analysis used a Q Exactive PLUS Hybrid Quadrupole-Orbitrap mass spectrometer (Thermo Fisher Scientific) using hydrophilic interaction chromatography. LC separation included the Dionex UltiMate 3000 UHPLC system (Thermo Fisher Scientific) with XBridge BEH amide column (Waters, Milford, MA) and XP VanGuard Cartridge (Waters, Milford, MA). LC gradients were as follows: solvent A (95%:5% H_2_O:acetonitrile with 20 mM ammonium acetate, 20 mM ammonium hydroxide, pH 9.4); solvent B (20%:80% H_2_O:acetonitrile with 20 mM ammonium acetate, 20 mM ammonium hydroxide, pH 9.4); solvent B percentages over time: 0 min, 100%: 3 min, 100%; 3.2 min, 90%; 6.2 min, 90%; 6.5 min, 80%; 10.5 min, 80%; 10.7 min, 70%; 13.5 min, 70%; 13.7 min, 45%; 16 min, 45%; 16.5 min, 100%. Flow rate was 300 µL/min and injection volume 5 µL and temperature maintained at 25°C. MS scans used negative ion mode, resolution of 70,000 at *m/z* 200, and an automatic gain control target of 3 × 10^6^ and scan range of 72–1,000. MAVEN software package was used to analyze metabolite data (43).

### Immunoblotting

Western blots were performed using standard lab procedures. Log phase cultures were lysed in SDS-PAGE loading buffer and boiled for 10 min. Equivalent OD units of cell lysate were loaded. Standard procedures were followed for SDS-PAGE and protein transfer to nitrocellulose membrane. Antibodies were used at the following concentrations: Primary antibodies used were Flag-M2—1:1,000 (Sigma, St. Louis, MO); MreB—1:10,000 (Régis Hallez, University of Namur); MipZ—1:5,000 (30); FtsZ—1:20,000 (36); and CdnL—1:10,000 (42). Secondary antibodies used were 1:10,000 of HRP-labeled α-mouse (for Flag) (PerkinElmer) or α-rabbit (PerkinElmer) (for MreB, MipZ, FtsZ, and CdnL). Chemiluminescent substrate (PerkinElmer) was added to visualize proteins via an Amersham Imager 600 RGB gel and membrane imager (GE).

## SUPPLEMENTAL MATERIAL

Supplemental Material mbio.01443-24-s0001.docx Supplemental figures and legends.

Table S1 mbio.01443-24-s0002.xlsx Metabolomic analysis of OpgH depleted cells.

Table S2 mbio.01443-24-s0003.xlsx Strains and plasmids used in this study.

ASM does not own the copyrights to Supplemental Material that may be linked to, or accessed through, an article. The authors have granted ASM a non-exclusive, world-wide license to publish the Supplemental Material files. Please contact the corresponding author directly for reuse.
