# Supplementary material for: OpgH is an essential regulator of Caulobacter morphology
Source: mBio. 2024 Aug 15;15(9):e01443-24. doi: 10.1128/mbio.01443-24 (PMC11389396; doi:10.1128/mbio.01443-24)
Supplement: Supplemental Material — Supplemental figures and legends. [file mbio.01443-24-s0001.docx]

**
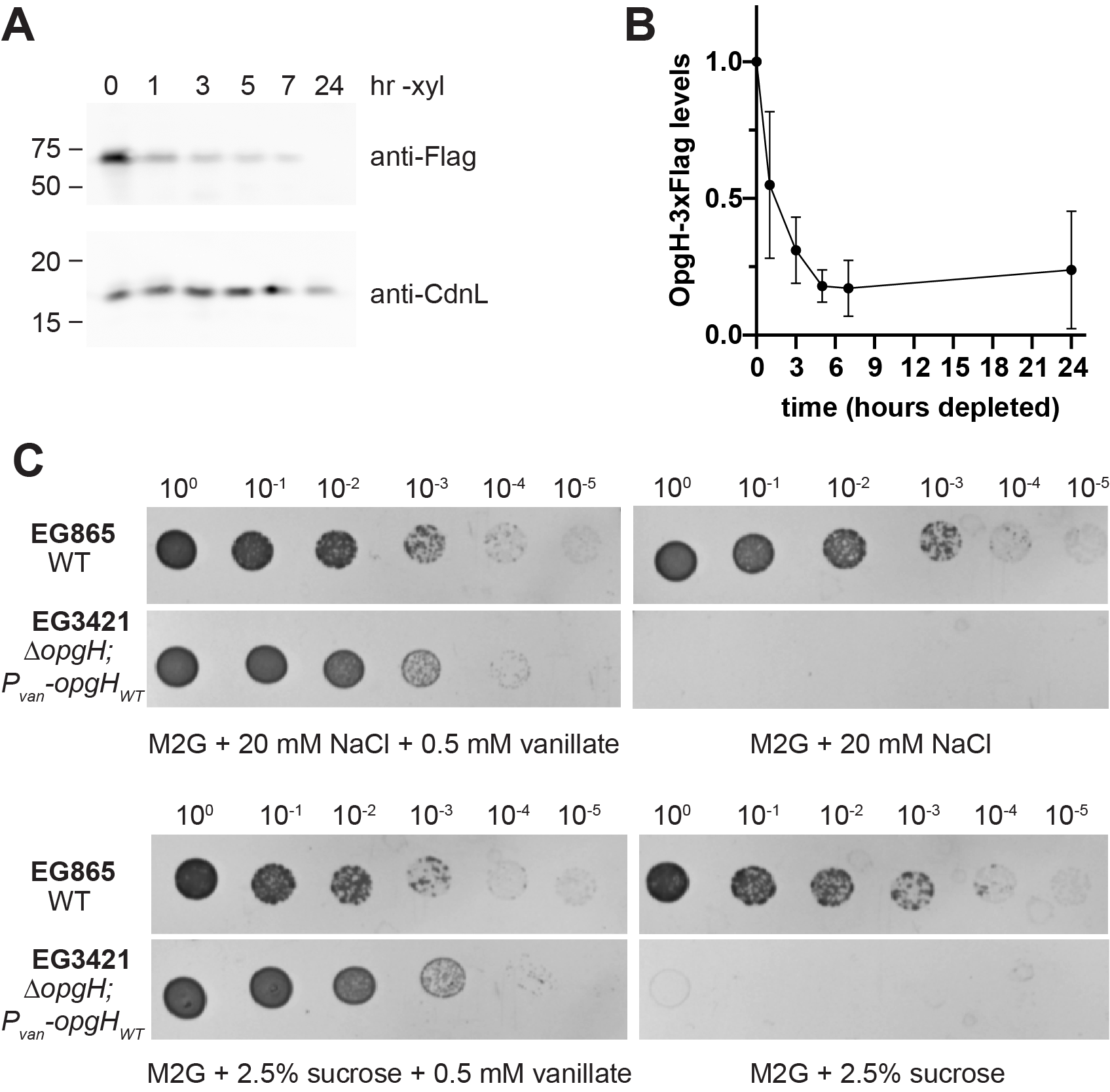
**

**Supplemental Figure 1**.

The OpgH depletion phenotype is reliant on cell growth and is lethal in high osmolarity.

**A.** Representative immunoblot of EG3957 over 24 hours of OpgH-3xFlag depletion. CdnL was used as a loading control. **B.** Corresponding densitometry analysis of OpgH-3xFlag depletion for the immunoblot example in **(A)**. Levels were normalized to total protein and plotted relative to t= 0. Error bars indicate ± 1 standard deviation for 3 biological replicates. **C.** Spot dilutions of WT (EG865) and the OpgH depletion strain (EG3421; ∆*opgH* + P_van_-*opgH*) grown on M2G with high salt (20 mM NaCl) or high sucrose (2.5% w/v) with or without vanillate induction for two days.


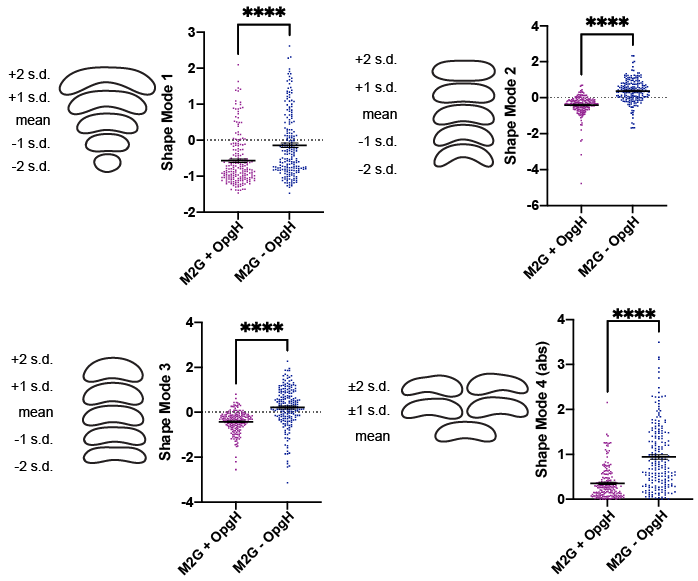


**Supplemental Figure 2.**

OpgH depletion causes morphological defects in minimal media

Principal component analysis (PCA) of the OpgH depletion strain (EG3421) after 5 hours grown in M2G with 0.5 mM vanillate (purple, +OpgH) or without vanillate (blue, -OpgH). Scatter plots of 200 cells are presented. Shape modes 1, 2, 3, and 4 correspond to length, curvature, width, and asymmetric bulging. Contours indicate the mean shape and 1 or 2 standard deviations from the mean. Shape mode 4 shows the absolute value. Statistical analysis uses a Mann-Whitney unpaired t-test. **** = P < 0.0001.


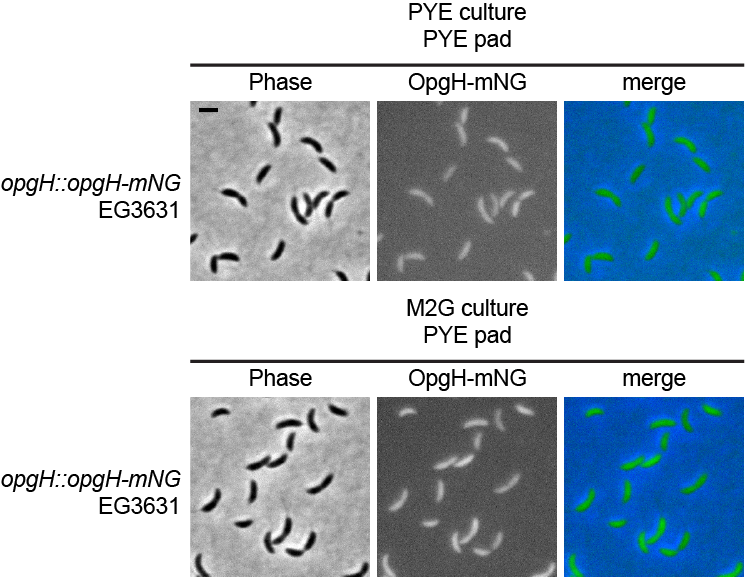


**Supplemental Figure 3.**

OpgH is diffuse within the cell.

Phase contrast, epifluorescence, and merged images showing localization of OpgH with a C-terminal mNeonGreen (mNG) tag (EG3631) grown in PYE or M2G. Scale bar (2 µm) applies to every image.


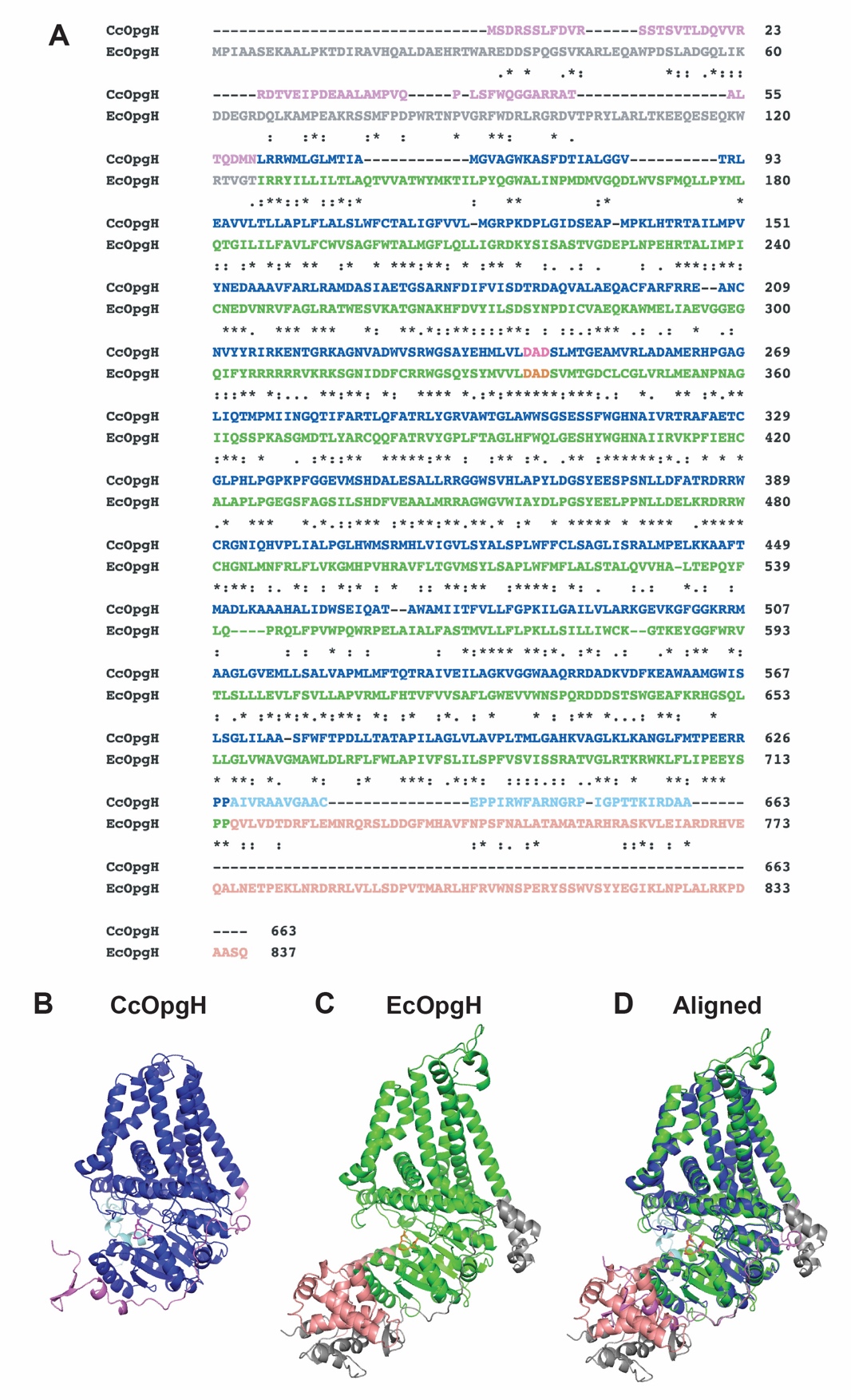


**Supplemental Figure 4.**

Sequence and structure alignments of *E. coli* and *Caulobacter* OpgH reveal similarities and differences.

**A**. The primary sequences of *Caulobacter* (CcOpgH) and *E. coli* (EcOpgH) were aligned Clustal Omega. The poorly conserved N- and C-terminal regions of each are colored independently from the well-conserved central region. The predicted catalytic D-A-D residues of each are highlighted in magenta (CcOpgH) and orange (EcOpgH). **B** and **C**. Alphafold predicted structures of CcOpgH (B) and EcOpgH (C) with residues colored as in (A) and oriented with the predicted periplasmic face at the top and cytoplasmic regions at the bottom. **D.** The structures in B and C were aligned in Pymol yielding an rmsd of 0.678. Major differences observed are in the N- and C-termini, as well as in predicted periplasmic loops of EcOpgH.


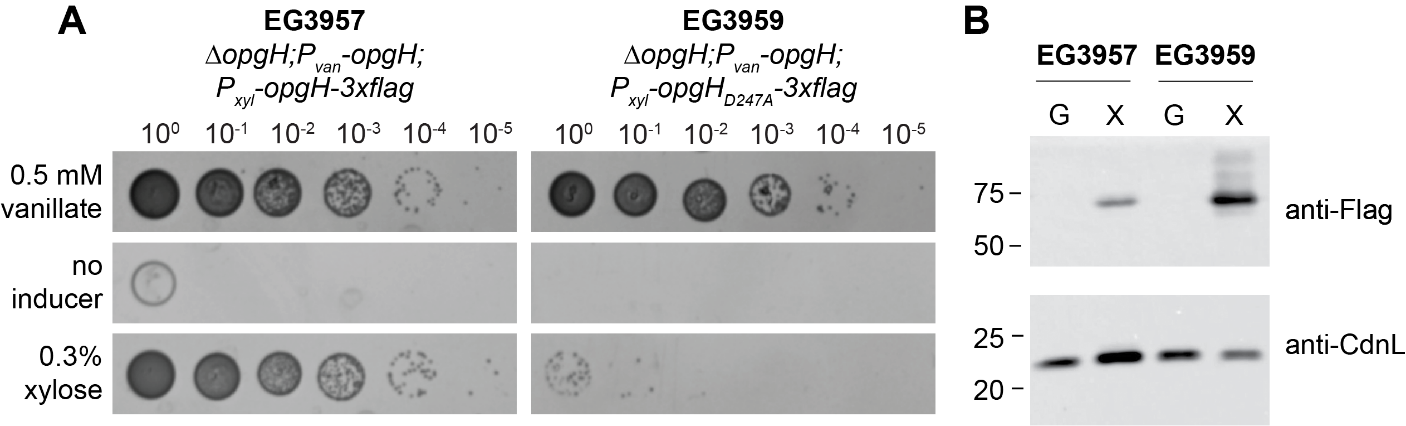


**Supplemental Figure 5.**

3x-Flag-tagged OpgH_D247A_ cannot complement loss of OpgH but is stably produced.

**A.** Spot dilutions of the OpgH depletion strain (∆*opgH* + P_van_-*opgH*) with xylose inducible WT *opgH-3xfllag* (EG3957) or *opgH_D247A_-3xfllag* (EG3959) grown on M2G with indicated inducer for two days. **B.** Immunoblot of lysates from the indicated strains grown in PYE with glucose (G) or xylose (X) for 6.5 hours. CdnL was used as a loading control.


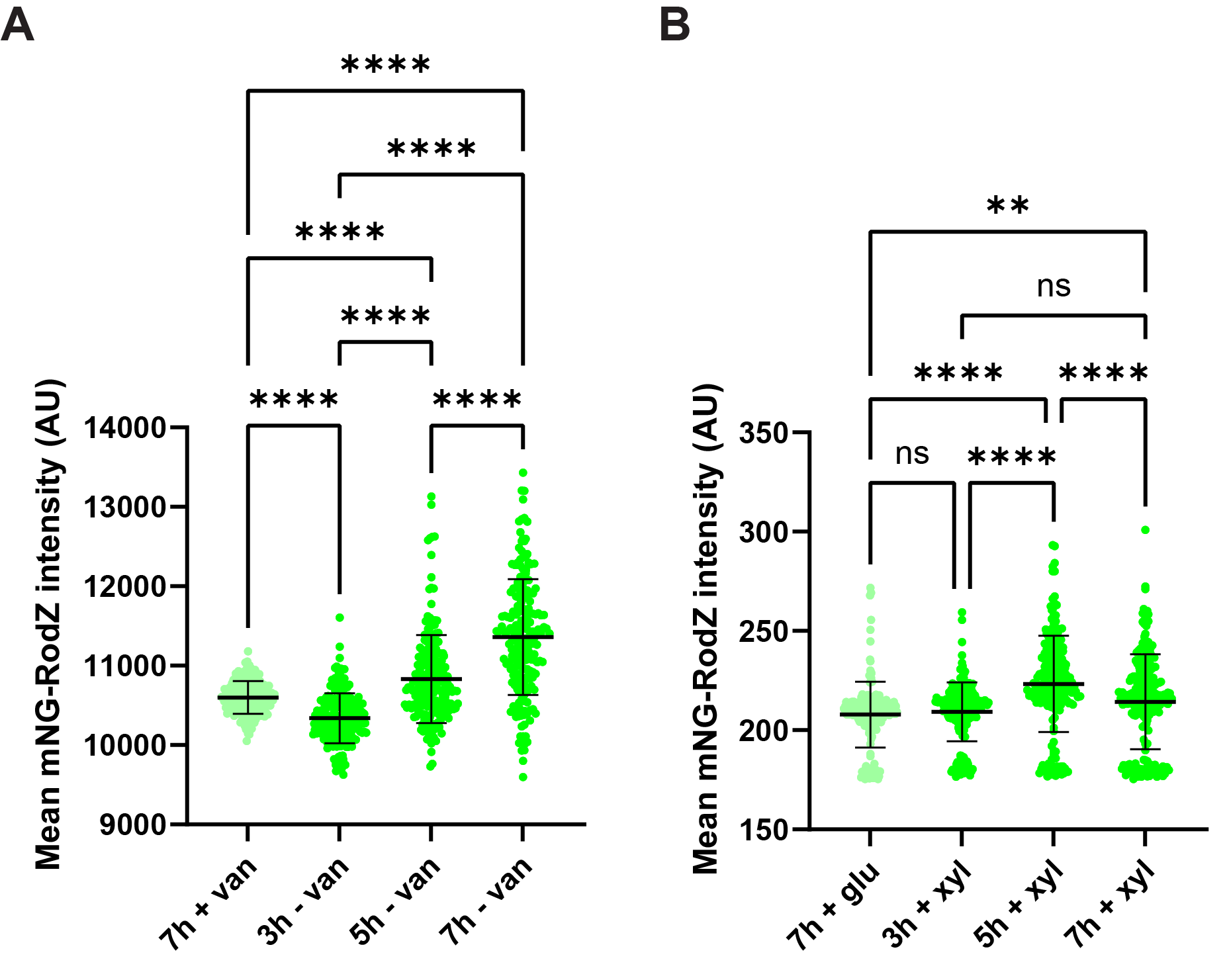


**Supplemental Figure 6.**

mNG-RodZ fluorescence increases during OpgH depletion and *cenR* overexpression.

Cells were **(A)** depleted of OpgH (EG3790) by vanillate removal (- van) or **(B)** induced with xylose (+ xyl) for *cenR* overexpression (EG3990). Seven hour induced OpgH (+ van) or uninduced *cenR* glucose (+ glu) controls, respectively, were used. mNG-RodZ mean fluorescence intensity for 210 cells per time point per strain was measured using MicrobeJ. Center line indicates mean and error bars indicate ± 1 standard deviation. Statistical analysis uses ANOVA comparing each mean to every other mean. ns = not significant; ** = p < 0.001; **** = p < 0.0001


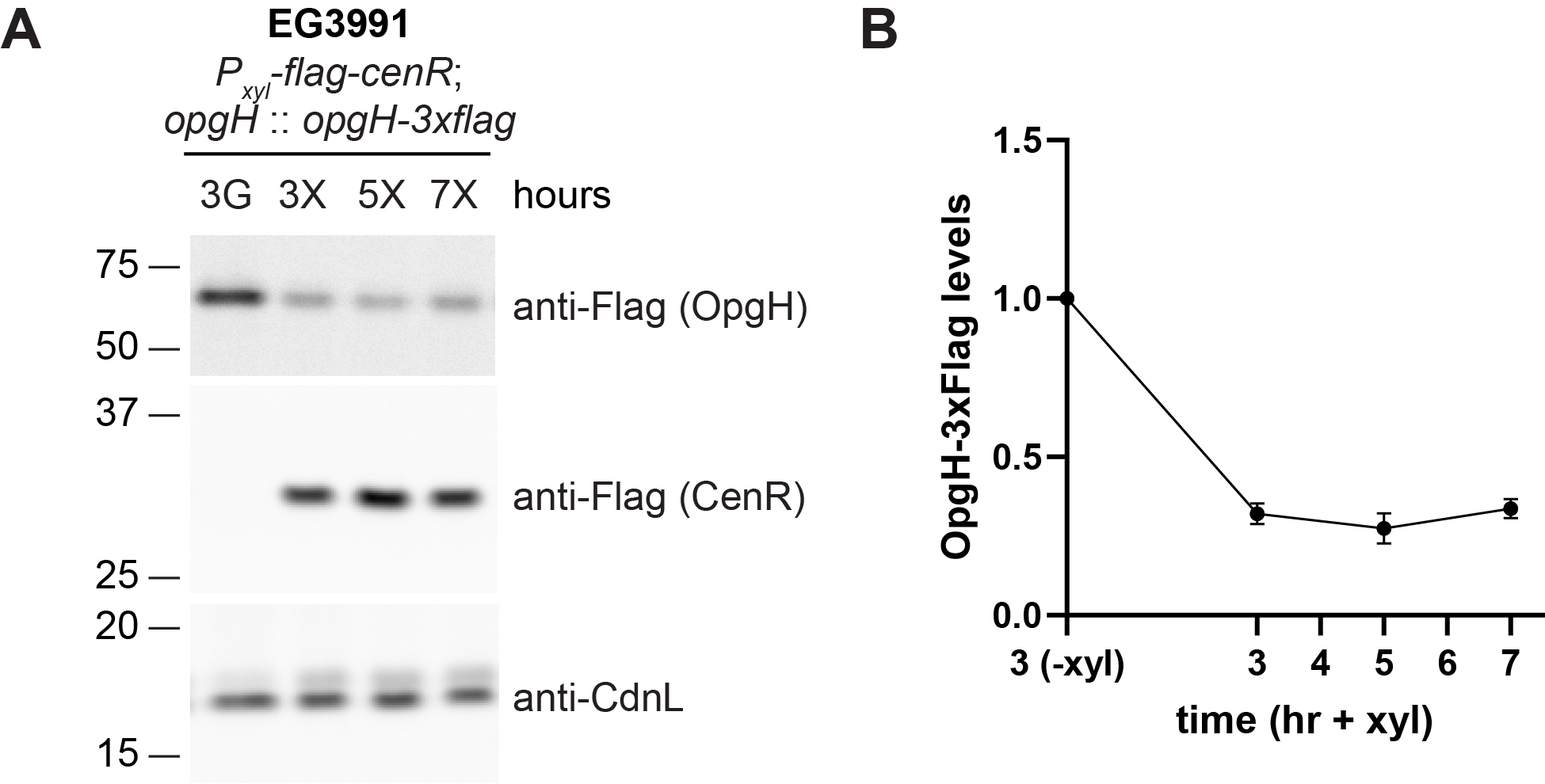


**Supplemental Figure 7**

OpgH levels decrease during *cenR* overexpression.

**A.** Representative immunoblot and **(B)** corresponding densitometry analysis of OpgH-3xFlag levels over 7 hours of *flag-cenR* overexpression using 0.3% xylose. CdnL was used as a loading control. Levels were normalized to total protein and plotted relative to 3 hours uninduced *cenR* (3 - xyl). Error bars indicate ± 1 standard deviation for 3 biological replicates.


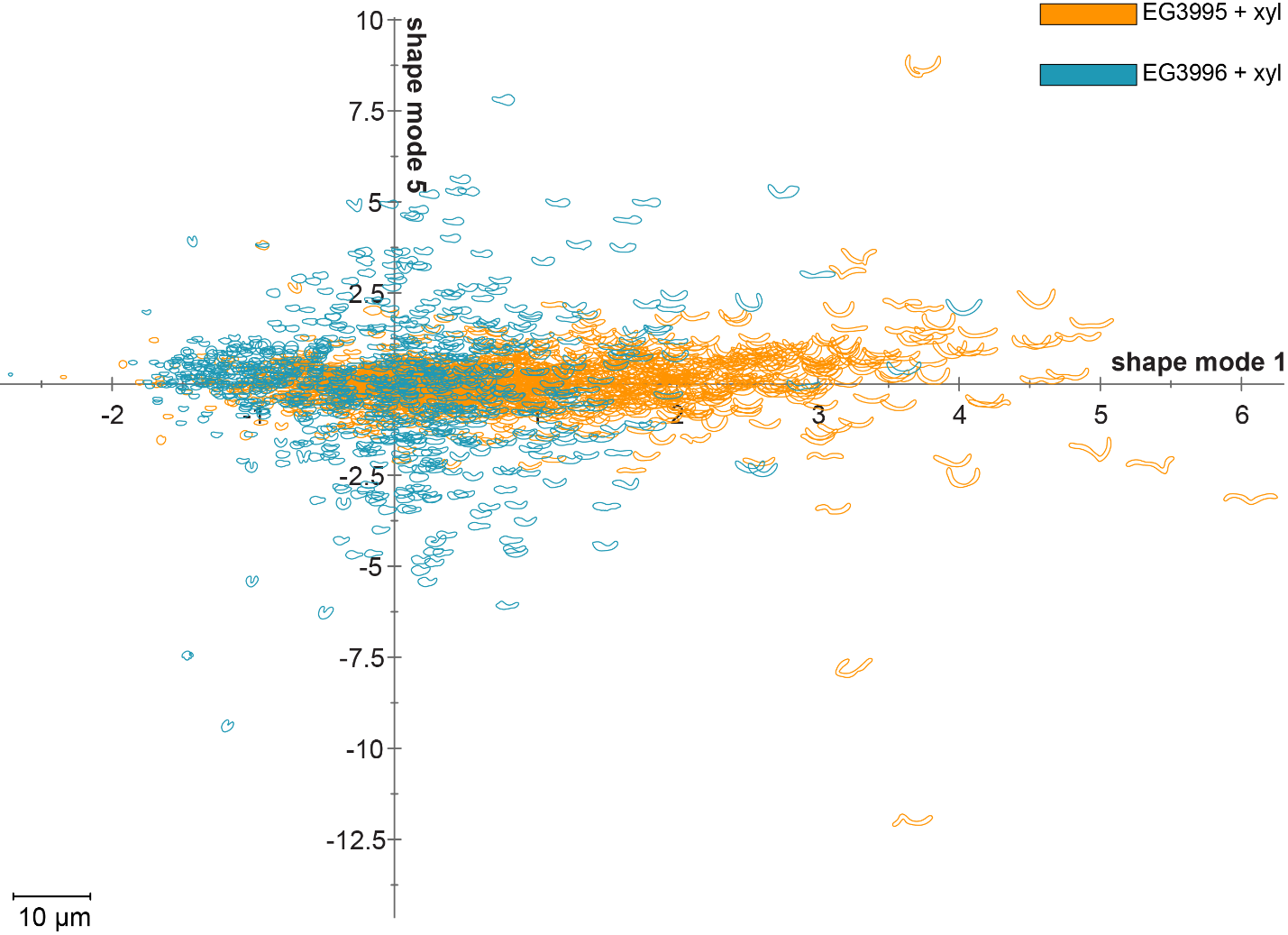


**Supplemental Figure 8**

Cells overexpressing *cenR* and *opgH* are elongated but do not bulge.

Plot comparing shape mode 5 (asymmetric bulging) to shape mode 1 (length) after 7 hours of xylose overexpression (+ xyl) of *cenR* with WT *opgH* (EG3995, orange) or with *opgH_D247A_* (EG3996, blue). For the three EG3995 + xyl replicates, 535, 453, and 468 cells were used. For EG3996 + xyl, 347, 317, and 161 cells were used. Plot was made using CellTool.

**Supplemental Table 1.**

Polar metabolites in extracts from cells producing OpgH or depleted of OpgH for 5 h in M2G or PYE. Metabolites reduced at least two-fold during OpgH depletion in both media conditions are highlighted in red, those increased at least two-fold during OpgH depletion in both media conditions are highlighted in green.

**Supplemental Table 2.**

Strains and plasmids used in this study.
